# The BACE1-generated C-terminal fragment of the neural cell adhesion molecule 2 (NCAM2) promotes BACE1 targeting to Rab11-positive endosomes

**DOI:** 10.1007/s00018-022-04575-w

**Published:** 2022-10-17

**Authors:** Ryan Keable, Shangfeng Hu, Grant Pfundstein, Irina Kozlova, Feifei Su, Ximing Du, Hongyuan Yang, Jenny Gunnersen, Melitta Schachner, Iryna Leshchyns’ka, Vladimir Sytnyk

**Affiliations:** 1 School of Biotechnology and Biomolecular Sciences, The University of New South Wales, Sydney, NSW 2052 Australia; 2 Department of Anatomy and Physiology, School of Biomedical Sciences, Faculty of Medicine, Dentistry and Health Sciences, The University of Melbourne, Parkville, VIC 3010 Australia; 3 Department of Cell Biology and Neuroscience, Keck Center for Collaborative Neuroscience, Rutgers University, Piscataway, NJ 08554 USA

**Keywords:** Cell adhesion, BACE1, Neurons, Recycling endosomes, Rab11, Proteolysis, Shedding

## Abstract

Beta-site amyloid precursor protein cleaving enzyme 1 (BACE1), also known as β-secretase, is an aspartic protease. The sorting of this enzyme into Rab11-positive recycling endosomes regulates the BACE1-mediated cleavage of its substrates, however, the mechanisms underlying this targeting remain poorly understood. The neural cell adhesion molecule 2 (NCAM2) is a substrate of BACE1. We show that BACE1 cleaves NCAM2 in cultured hippocampal neurons and NCAM2-transfected CHO cells. The C-terminal fragment of NCAM2 that comprises the intracellular domain and a small portion of NCAM2’s extracellular domain, associates with BACE1. This association is not affected in cells with inhibited endocytosis, indicating that the interaction of NCAM2 and BACE1 precedes the targeting of BACE1 from the cell surface to endosomes. In neurons and CHO cells, this fragment and BACE1 co-localize in Rab11-positive endosomes. Overexpression of full-length NCAM2 or a recombinant NCAM2 fragment containing the transmembrane and intracellular domains but lacking the extracellular domain leads to an increase in BACE1 levels in these organelles. In NCAM2-deficient neurons, the levels of BACE1 are increased at the cell surface and reduced in intracellular organelles. These effects are correlated with increased levels of the soluble extracellular domain of BACE1 in the brains of NCAM2-deficient mice, suggesting increased shedding of BACE1 from the cell surface. Of note, shedding of the extracellular domain of Sez6, a protein cleaved exclusively by BACE1, is reduced in NCAM2-deficient animals. These results indicate that the BACE1-generated fragment of NCAM2 regulates BACE1 activity by promoting the targeting of BACE1 to Rab11-positive endosomes.

**Supplementary Information:**

The online version contains supplementary material available at 10.1007/s00018-022-04575-w.

## Introduction

The neural cell adhesion molecule 2 (NCAM2) is a cell surface glycoprotein of the immunoglobulin superfamily of cell adhesion molecules [1]. Two NCAM2 isoforms, generated via alternative splicing, have identical extracellular domains comprising five immunoglobulin-like (Ig) and two fibronectin type III (Fn) repeats but differ in their membrane anchorage. The shorter isoform is glycosylphosphatidylinositol anchored, whereas the longer isoform is a transmembrane protein with an intracellular domain of 119 amino acids [2–4]. During development, NCAM2 promotes neurite outgrowth as well as branching and guidance of dendrites and axons of differentiating neurons [5–8]. NCAM2 also contributes to maturation of developing synapses [9]. In the mature brain, NCAM2 accumulates in excitatory synapses, where it forms trans-synaptic homophilic adhesive bonds involved in synapse maintenance [10, 11]. Deletions of the *NCAM2* gene are found in individuals with neurodevelopmental disorders, intellectual disability, and autism and other deficits [12–14]. NCAM2 is encoded by a gene located on chromosome 21 in humans, which is triplicated and thereby overexpressed in Down’s syndrome [15]. The overall levels of NCAM2 are increased in sporadic Alzheimer’s disease (AD), whereas its synaptic levels are reduced [11]. Single nucleotide polymorphisms (SNPs) in the *NCAM2* gene are associated with an increased risk of late-onset AD [16]. SNPs in the *NCAM2* gene are also associated with increased cerebrospinal fluid levels of Aβ, an insoluble toxic peptide accumulating in AD brains [17], suggesting that NCAM2 is involved in regulating the expression and activity of proteolytic enzymes involved in amyloid β (Aβ) production.

One such proteolytic enzyme is the beta-site amyloid precursor protein cleaving enzyme 1 (BACE1). This aspartic acid protease mediates a rate-limiting step in Aβ production. BACE1 activity is highest in the acidic environment of endosomes. To reach endosomes, BACE1 is transported via the Golgi apparatus and Golgi-derived vesicles to the cell surface [18, 19] from where BACE1 is endocytosed via a clathrin-dependent pathway to reach early and Rab11-positive recycling endosomes [18–20]. BACE1 then shuttles between the plasma membrane and the endosomal system [18]. BACE1 can also be targeted from the endosomal system to lysosomes for degradation [21] or retrogradely transported to the trans-Golgi network [22]. Trafficking of newly synthesized BACE1 is polarized in neurons, where it is initially targeted to the cell surface of dendrites, wherefrom it is endocytosed into early and recycling endosomes [23–25]. From dendrites, BACE1 redistributes to axons and synaptic terminals via the cell soma, a process in which Rab11-positive slowly recycling endosomes appear to play a major role [24, 26]. In contrast, BACE1-containing early endosomes are stationary in dendrites [27]. Rab11-dependent transport of BACE1 is required for its activity towards some of its substrates, including amyloid precursor protein (APP) [25, 28]. The removal of BACE1 from synaptic terminals occurs via dynein-snapin-mediated retrograde axonal transport [26]. While the role of endosomes in the axonal trafficking of BACE1 and its activity towards APP is well established, mechanisms regulating BACE1 targeting to the recycling endosomes are less understood. The activity of BACE1 towards its substrates can also be regulated via the shedding of the ectodomain of BACE1 from the plasma membrane [29, 30], since soluble BACE1 is unable to cleave APP [31]. Mechanisms regulating BACE1shedding by proteolysis remain also mostly unknown.

BACE1 cleaves NCAM2 in the second Fn domain at amino acids 662–663, and this cleavage results in the release of a part of the extracellular domain of NCAM2 from the cell surface and the formation of a C-terminal fragment comprising the whole intracellular and transmembrane domain in conjunction with the residual 35 amino acids of the extracellular domain of NCAM2 [32]. In the present study, we show that the BACE1-generated C-terminal fragment of NCAM2 associates with BACE1 and regulates the targeting of BACE1 to Rab11-positive endosomes. NCAM2 deficiency leads to the accumulation of BACE1 at the dendritic cell surface and enhances the shedding of BACE1. Our results suggest that the cleavage of NCAM2 by BACE1 represents a yet unknown mechanism by which BACE1 can be sorted to recycling endosomes.

## Materials and methods

### Antibodies and inhibitors

Goat polyclonal antibodies against the C-terminal intracellular amino acids 822–836 of human NCAM2 (14 of 15 amino acids are conserved in mouse NCAM2) from GeneTex (cat# GTX89311) were used in the proximity ligation (PL) assay (PL, 1:100), immunocytochemistry (IC, 1:100), and Western blot (WB, 1:1000). The mouse monoclonal antibody recognizing an extracellular epitope located within the first Fn repeat of mouse NCAM2 (Supplementary Fig. S1) from Santa Cruz Biotechnology (cat# sc-136328) was used in IC (1:100) and WB (1:1000). The specificity of these antibodies had been confirmed [9, 11] and herein by using NCAM2−/− tissue (Fig. 9B). The rabbit monoclonal antibody against the intracellular domain of BACE1 (D10E5, cat# 5606) from Cell Signaling Technology [33] was used for PL (1:100). Rabbit polyclonal antibodies against the N-terminus of BACE1 (amino-acids 38–70 of human BACE1, cat# AP14566PU-N) from OriGene were used for IC (1:100). The mouse monoclonal antibody against synaptophysin (cat# sc-17750, IC at 1:100) was from Santa Cruz Biotechnology. The mouse monoclonal antibody against the FLAG tag (cat# F1804, IC at 1:1000), rabbit polyclonal antibodies against the human influenza hemagglutinin (HA) tag (cat # H6908, IC at 1:100), mouse monoclonal antibody against glyceraldehyde 3-phosphate dehydrogenase (GAPDH, cat # G8795, WB at 1:1000), and mouse monoclonal antibody against actin (cat# A1978, WB at 1:3000) were from Sigma-Aldrich. The mouse monoclonal antibody against Rab11 (cat# 610656, IC at 1:50, WB at 1:1000), mouse monoclonal antibody against Rab4 (cat# 610888, WB at 1:1000), and mouse monoclonal antibody against Rab5 (cat# 610725, WB at 1:1000) were from BD Biosciences. β-Amyloid (1–42 specific) (D3E10) rabbit monoclonal antibodies (cat# 12843, Dot Blot at 1:2000) were from Cell Signaling Technology. Rabbit polyclonal antibodies against Sez6 were as described [34]. Fluorochrome- and HRP-conjugated secondary antibodies were from Jackson ImmunoResearch. BACE1 inhibitor (β-Secretase Inhibitor IV, cat# sc-222304), which inhibits BACE1 (IC_50_ = 15 nM) and BACE2 (IC_50_ = 0.23 µM), was from Santa Cruz Biotechnology. Ethylenediaminetetraacetic acid (EDTA)-free complete inhibitors were from Roche.

### DNA constructs and small interfering RNAs (siRNAs)

Bace1 (Myc-Flag-tagged, cat# MR208042) and Bace2 (Myc-Flag-tagged, cat# MR220631) were from OriGene. HA-tagged full-length transmembrane isoform of human NCAM2 was as described [11] and used as a template to produce HA-tagged NCAM2ΔED comprising transmembrane and intracellular domains of NCAM2 (aa 694–837). pcDNA3 was from Life Technologies. BACE1 siRNA (cat# sc-37225), BACE2 siRNA (cat# sc-29777) and control siRNA (cat# sc-37007) were from Santa Cruz Biotechnology. The efficiency of knock-down was verified in transfected CHO cells and cultured hippocampal neurons (Supplementary Fig. S2). BACE1 fused to the C-terminal amino acid residues 155–238 of the Venus fluorescent protein (BACE1-VC) was a kind gift of Subhojit Roy [35]. Rab11-DsRed (Addgene, plasmid # 12679 [36]), DsRed-Golgi [37], Lamp1-EYFP [37], and GFP-Sec61B (Addgene, plasmid # 121159 [38]) were as described. mTagBFP-Nucleus-7 (Addgene, plasmid # 55265) was a kind gift of Michael Davidson (unpublished). Cherry was a kind gift from Roger Tsien [39]. DNA constructs coding for Ig1, Ig2, Ig3, Ig4, Ig5, Fn1, Fn2 and intracellular domain (ID) of NCAM2 were synthesized and subcloned into the pET100 vector using GeneArt services (Thermo Fisher Scientific). HA-NCAM2-VN and HA-NCAM2ΔED-VN were generated by inserting the first 155 amino acids of Venus (VN) at the C-terminus of HA-NCAM2 and HA-NCAM2ΔED, respectively, with an intervening 10 amino acid linker (PRARDPPVAT). Briefly, DNA encoding VN bearing an I152L point mutation was PCR amplified from pBiFC-VN155(I152L) (Addgene, plasmid # 27097 [40]) and inserted at the extreme C-terminal end of NCAM2 using the NEBuilder HiFi DNA Assembly kit (New England Biolabs) according to the manufacturer’s instructions. Both constructs were cloned in frame and fidelity confirmed by sequencing.

### Animals

NCAM2-deficient mice (STOCK Ncam2tm1Mom/MomJ; Stock No: 006706) were from the Jackson Laboratory. For biochemical analyses of adult 2-month-old brain tissue and dot blot analysis of Aβ1-42 levels in 5–6-month-old brains, NCAM2+/+, NCAM2+/− and NCAM2−/− littermates from heterozygous breeding pairs were used. 0- to 3-day-old NCAM2 + / + and NCAM2−/− mice for cell culture and biochemical analysis were obtained using homozygous breeding pairs of respective genotypes. We also used C57BL/6 mice in experiments, which were not aimed at comparing mice of different genotypes. Experiments were approved by the Animal Care and Ethics Committee of the University of New South Wales (permit 18/99A (for work with C57BL/6 mice) and 17/102A & 20/93B (for work with transgenic mice)).

### Neuronal cell culture and transfection

Mouse hippocampal neurons were prepared from 0- to 3-day-old NCAM2 + / + and NCAM2−/− mice or C57BL/6 mice and cultured as described [9, 41]. Neurons were maintained in Neurobasal A supplemented with 2% B-27, glutamine, and 2 ng/ml FGF-2 on glass coverslips coated with poly-d-lysine (100 μg/ml) (all reagents were from Thermo Fisher Scientific). Neurons were transfected before plating by electroporation using a Neon transfection system (Thermo Fisher Scientific). Alternatively, neurons were transfected using the calcium phosphate method essentially as described [42]. Briefly, coverslips with neurons maintained for 7 to 10 days in culture were transferred 30 min prior to transfection to a 24-well-plate containing 500 µl/well culture medium. One µg of plasmid DNA and 3.1 µl of 2 M CaCl_2_ were mixed with water to a final volume of 25 µl per coverslip. DNA-Ca^2+^-phosphate precipitate was prepared by adding DNA/CaCl_2_ solution to 25 µl of 2 × HEPES-buffered saline (HBS, 280 mM NaCl, 1.5 mM Na_2_HPO_4_, 50 mM HEPES, pH 7.10) (1/8th at a time and mixing briefly between each addition). The solution was incubated for 10 min at 37 °C and the resulting suspension was applied dropwise to the coverslips. Neurons were incubated with the precipitate for 3 h, washed with acidified Tyrode’s solution (pH 6.7–6.8) to remove the precipitate, and transferred back to the wells containing the original conditioned culture medium.

### CHO cell culture and transfection

CHO cells were maintained in Dulbecco’s Modified Eagle’s Medium/Nutrient Mixture F-12 HAM (Sigma) supplemented with 5% newborn calf serum (Sigma) in an incubator at 37 °C and 5% CO_2_. For immunofluorescence analysis, CHO cells were plated on glass coverslips in a 24-well-plate in a culture medium and transfected using Lipofectamine 3000 transfection reagent (Thermo Fisher Scientific) according to the manufacturer’s instructions. Alternatively, cells were transfected using the calcium phosphate method as described in *Neuronal culture and transfection.* Neuronal cultures were supplied with fresh culture medium prior to transfection, and CHO cells were treated with culture medium without serum. After transfection, neurons were washed with Tyrode’s solution, and CHO cells were treated with 15% glycerol dissolved in phosphate-buffered saline (PBS) for 1.5 min, washed with PBS and supplied with fresh culture medium containing 5% serum. Cells were incubated for 24 h with BACE1 inhibitor IV (30 nM), dynasore (80 µM [39]) or vehicle (0.1% DMSO in culture medium) at 24 h after transfection.

### Immunofluorescence labeling of BACE1 at the cell surface of cultured cells

Live CHO cells and neurons were incubated with rabbit polyclonal antibodies against the N-terminus of BACE1 diluted in culture medium for 20 min on ice, fixed with 4% formaldehyde in PBS for 15 min, washed with PBS, blocked with 1% donkey serum diluted in PBS and incubated with fluorochrome conjugated anti-rabbit secondary antibodies for 45 min at room temperature. Cells were then washed with PBS and postfixed in 4% formaldehyde in PBS for 5 min at room temperature. Fixed cells were immunolabeled as described in “Immunofluorescence labeling of fixed cultured cells”.

### Immunofluorescence labeling of cultured cells

The labeling was done essentially as described [43]. In brief, cultured neurons and CHO cells were fixed with 4% formaldehyde in PBS for 15 min at room temperature and washed with PBS. Cells were permeabilized with 0.25% Triton X-100 in PBS for 5 min and blocked with 1% donkey serum in PBS for 20 min at room temperature. Cells were then incubated with primary antibodies at 4 °C overnight, washed with PBS and incubated with fluorochrome-conjugated secondary antibodies for 45 min at room temperature. All antibodies were diluted in PBS containing 1% donkey serum. Thereafter, cells were embedded in ProLong Gold Antifade Mountant (Thermo Fisher Scientific).

### Analysis of BACE1 endocytosis in cultured neurons and CHO cells

Cells were incubated with rabbit polyclonal antibodies against the N-terminus of BACE1 diluted in culture medium for 30 min in the incubator, washed with PBS, fixed with 4% formaldehyde in PBS for 15 min at room temperature, washed again with PBS, blocked with 1% donkey serum diluted in PBS for 20 min at room temperature and incubated with fluorochrome-conjugated anti-rabbit secondary antibodies for 1 h at room temperature to detect cell surface BACE1. To detect endocytosed BACE1–BACE1 antibody complexes, cells were then washed with PBS, postfixed with 2% formaldehyde in PBS for 5 min at room temperature, washed with PBS, permeabilized with 0.25% Triton X-100 for 5 min, blocked with 1% donkey serum diluted in PBS for 20 min at room temperature and incubated with anti-rabbit secondary antibodies conjugated to a fluorochrome, which differed from the one used to detect cell surface BACE1, for 45 min at room temperature. All secondary antibodies were diluted in PBS containing 1% donkey serum. Labeling for other antigens was done as described in “*Immunofluorescence labeling of fixed cells*”.

### Proximity ligation assay (PL assay)

The assay was performed essentially as described [44]. Cultured neurons were fixed in 4% formaldehyde in PBS, washed with PBS, permeabilized with 0.25% Triton X-100 in PBS for 5 min, and blocked with 1% bovine serum albumin (BSA) in PBS for 20 min. Antibodies against the intracellular domain of NCAM2 and BACE1 were applied to the cells in 0.1% BSA in PBS overnight at 4 °C. Further steps were performed using secondary antibodies conjugated with oligonucleotides (PL assay probes, Olink Bioscience, Uppsala, Sweden) and Duolink II fluorescence kit (Olink Bioscience) in accordance with the manufacturer’s instructions.

### Confocal microscopy and image analysis

Fluorescence images were acquired at room temperature using the confocal laser scanning microscope Nikon C1si, NIS Elements software and CFI Plan Apochromat VC 60XH objective (numerical aperture 1.4) all from Nikon Corporation (Tokyo, Japan). Image analysis was done with ImageJ.

### Analysis of the density of intracellular BACE1 clusters in cultured neurons

Surface and total BACE1 clusters/puncta were outlined using the threshold function, with a constant threshold set to 4 times the average mean intensity along dendrites in each channel. To count intracellular puncta, a mask of all surface labeling above the threshold was subtracted from the images for determination of the total BACE1 labeling. Thereafter, the remaining dendritic puncta, which were not detectable at the cell surface, were counted using the analyze particles tool in ImageJ. The numbers were normalized to dendritic length to obtain the density of intracellular BACE1 puncta.

### Analysis of BACE1 targeting to organelles in CHO cells

Organelles were outlined using the threshold function of ImageJ. The enrichment of BACE1 in these organelles was calculated by dividing the mean intensity of BACE1 labeling within the outlined organelles by the mean intensity of BACE1 labeling in the entire cell. The percentage of BACE1 labeling in organelles was calculated by dividing the integrated density of BACE1 labeling within the outlined organelles by the integrated density of BACE1 labeling within the entire cell.

### Analysis of BACE1 targeting to Rab11-positive endosomes in cultured hippocampal neurons

Rab11-positive endosomes were outlined using the threshold function in ImageJ. The enrichment of BACE1 was calculated by dividing the mean intensity of BACE1 labeling within the outlined endosomes by the mean intensity of BACE1 labeling in the entire dendrite. The percentage of BACE1 labeling in Rab11-positive endosomes was calculated by dividing the integrated density of BACE1 labeling in endosomes by the integrated density of BACE1 labeling in the entire dendrite.

### Preparation of brain homogenates, synaptosomes and soluble protein fractions

Brain tissue homogenates (10%, w/v) were prepared in HOMO-A buffer which was prepared from HOMO buffer (1 mM MgCl_2_, 1 mM CaCl_2_, 1 mM NaHCO_3_, 5 mM Tris, pH 7.4) and contained 0.32 M sucrose, EDTA-free complete inhibitors and 1 mM phenylmethylsulfonyl fluoride (PMSF). Homogenates were used for synaptosome isolation as described [39, 45]. All steps were performed at 4 °C. Briefly, homogenates were centrifuged at 1400*g* for 10 min. The supernatant and pellet were resuspended in HOMO-A buffer and centrifuged for 10 min at 700*g*. The resulting supernatants were pooled and centrifuged at 17,500*g* for 15 min. The supernatant was centrifuged at 200,000*g* for 1 h and used as the soluble fraction. The 17,500*g* pellet was resuspended in HOMO-A buffer and applied to the top of a step gradient with interfaces of 0.65 M, 0.85 M, 1.0 M, 1.2 M sucrose in HOMO buffer. The 700 g pellets were combined, adjusted to 1.0 M sucrose in HOMO buffer and layered onto 1.2 M sucrose in HOMO buffer. HOMO-A buffer was then applied to the top of the gradient. The crude synaptosomal fractions were collected at the 1.0 M/1.2 M interface after centrifugation for 2 h at 100,000 g and pooled. The crude synaptosomal fraction was again adjusted to 1.0 M sucrose and applied onto the 1.2 M sucrose cushion. HOMO-A buffer was then applied to the top of the gradient. After centrifugation for 2 h at 100,000*g*, synaptosomes were collected at the 1.0 M/1.2 M interface, resuspended in HOMO-A buffer, pelleted by centrifugation for 30 min at 100,000*g* and resuspended in HOMO-A buffer.

### Isolation of the transport vesicle-enriched fraction

Isolation of transport vesicles was performed essentially as described [46] (Fig. 2E). In brief, brain homogenates were prepared in 20 mM HEPES, 40 mM KCl, 5 mM EGTA, 5 mM EDTA, 1 mM PMSF and EDTA-free complete inhibitors. The homogenate was centrifuged at 1000*g* for 20 min at 4 °C. Pellet P1 was enriched in nuclei and plasma membranes. Supernatant S1 was then centrifuged at 10,200*g* for 20 min at 4 °C to produce crude synaptosomes (pellet P2). Supernatant S2 containing soluble proteins and transport vesicles was centrifuged at 100,000*g* for 1 h at 4 °C. The pellet P100 contained transport vesicles and supernatant S100 contained soluble proteins.

### Analysis of NCAM2 levels in neuronal culture medium and cell lysates

Hippocampal neurons were maintained in 6-well-plates for 14 days and then treated with β-Secretase Inhibitor IV (30 nM) or vehicle (0.1% dimethyl sulfoxide (DMSO)) for 24 h. Culture media were collected. Neurons were washed with ice-cold PBS and treated with lysis buffer (1% sodium deoxycholate, 1% Triton X-100, 50 mM Tris–Cl, 150 mM NaCl, 1 mM EDTA, EDTA-free complete inhibitors and 1 mM PMSF). Lysates were collected, incubated on ice for 30 min and centrifuged at 16,000*g* for 15 min at 4 °C. Supernatants were then collected for Western blot analysis. Culture media were centrifuged at 200,000*g* for 1 h at 4 °C, and the supernatants therefrom were collected. Proteins were precipitated on ice by adding 2% sodium deoxycholate to a final concentration of 0.03% and 20% trichloroacetic acid to a final concentration of 12% for 30 min. Precipitates were collected by centrifuging at 10,000*g* for 15 min at 4 °C, washed twice with ice-cold acetone, reconstituted in 50 µl lysis buffer and 50 µl 12 M urea, and used for Western blot analysis.

### Western blot analysis

Proteins and protein markers (Bio-Rad) were separated in 8% Bis–Tris bolt mini gel (Thermo Fisher Scientific) and electroblotted onto PVDF membranes (0.2 µm; Amersham). Membranes were washed with PBS, blocked with 5% skim milk in PBS, and incubated with appropriate primary antibodies overnight at 4 °C, followed by incubation with corresponding HRP-conjugated secondary antibodies for 1.5 h at room temperature. The antibodies were diluted in PBS containing 0.05% Tween. Protein bands were visualized by applying Luminata Forte Western HRP substrate (Merck Millipore) to the membrane. The chemiluminescence images were taken using Micro-Chemi 4.2 (DNR Bio-Imaging Systems) and analyzed with ImageJ.

### Dot blot analysis

Mouse brain homogenates or BSA as negative control were diluted in Tris-buffered saline (TBS), pH 7.4 and 10 µg of total protein was applied to a nitrocellulose membrane (0.2 µm, Bio-Rad). After drying for 15 min at room temperature, the membrane was stained with Ponceau to visualize total protein and then blocked using 5% skim milk in TBS. The membrane was then incubated overnight with rabbit monoclonal antibodies specific for Aβ42 (D3E10), followed by incubation with HRP-conjugated anti-rabbit secondary antibodies for 2 h at room temperature. Dots were visualized using Pierce ECL, imaged using Micro-Chemi 4.2 (DNR Bio-Imaging Systems) and analyzed with ImageJ.

### Statistical analysis

Unless indicated otherwise, all experiments were independently performed at least three times. Statistical analyses were done in GraphPad Prism 7. In experiments with immunofluorescence analysis, differences between two groups were analyzed using an unpaired *t* test, while differences between three or more groups were analyzed using one-way ANOVA and Dunnett’s multiple comparisons test. In each biochemical experiment, samples were organized into sets containing one sample of each kind, and each set was separately analyzed. Littermates were always included in the same set. Fold change relative to the level in the control sample was then calculated for each set, and the data from all sets were combined. One-sample *t* test was then used to estimate the statistical significance of the fold change relative to the control sample level.

## Results

### BACE1 is involved in the proteolytic processing of NCAM2 in hippocampal neurons

**Fig. 1:**
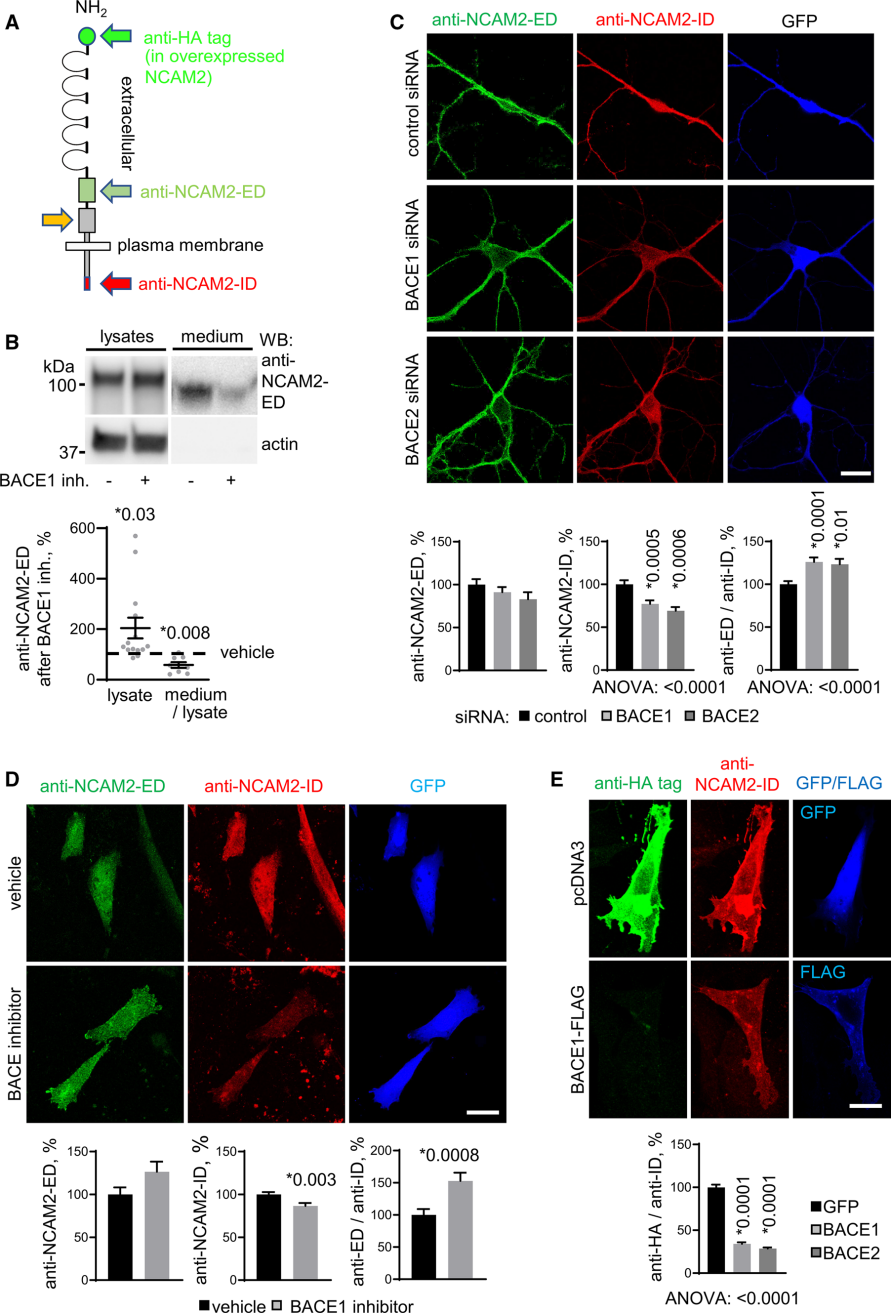
BACE1 is involved in proteolytic processing of NCAM2 in hippocampal neurons. **A** Schematic diagram showing locations of the domains recognized by antibodies against NCAM2-ED, NCAM2-ID, and HA tag (only in overexpressed NCAM2) used in this study. Orange arrow denotes the BACE1 cleavage site. **B** Lysates and medium from cultured hippocampal neurons treated with vehicle (0.1% DMSO) or BACE inhibitor analyzed by Western blot with anti-NCAM2-ED antibodies. Levels of NCAM2 are increased in lysates and reduced in the culture medium of neurons treated with the inhibitor. Graph shows NCAM2 levels in lysates (*n* = 14) and the ratios of medium/lysate levels (*n* = 8) from the inhibitor-treated neurons relative to levels in vehicle-treated neurons set to 100%. Means ± SEM are indicated. *p, one sample *t* test compared to the vehicle level. **C** NCAM2-ED and NCAM2-ID labeling in hippocampal neurons co-transfected with GFP and BACE1 siRNA, BACE2 siRNA or control siRNA. Bar = 20 μm. Graphs show mean + SEM NCAM2-ED and NCAM2-ID labeling intensities along dendrites of transfected neurons and their ratios (*n* > 46 neurons per group) normalized to the mean of control siRNA-transfected neurons set to 100%. *p, one-way ANOVA with Dunnett’s multiple comparisons test. **D** NCAM2-ED and NCAM2-ID labeling of CHO cells co-transfected with GFP and NCAM2 and treated with the BACE inhibitor or vehicle (0.1% DMSO). Bar = 20 μm. Graphs show mean + SEM NCAM2-ED and NCAM2-ID labeling intensities and their ratios (*n* > 173 cells per group) normalized to the mean of GFP-transfected cells set to 100%. *p, unpaired *t*-test. **E** NCAM2-ID and HA tag labeling in CHO cells co-transfected with HA-NCAM2 and GFP or BACE1-FLAG. Note the strongly reduced HA tag labeling in BACE1 co-transfected cells. Bar, 20 μm. Graph shows mean + SEM ratios of the HA tag and NCAM2-ID labeling intensities in CHO cells co-transfected with GFP, BACE1 or BACE2 (*n* > 188 cells per group) normalized to the means of GFP-transfected cells set to 100%. *p, one-way ANOVA and Dunnett’s multiple comparisons test

A recent report demonstrated that NCAM2 is cleaved by BACE1 at a membrane-proximal site within the second Fn repeat of the extracellular domain (ED) of NCAM2 in transfected HEK cells and in the olfactory bulb of mice (Fig. 1A, [32]). This study did not find changes in the levels of full-length NCAM2 or its cleavage products in the hippocampus of BACE1 knock-out mice. However, since BACE1 and NCAM2 are expressed in the hippocampus, the effect of BACE1 deficiency on NCAM2 processing could have been obscured by yet unidentified compensatory proteolytic mechanisms. To determine whether BACE1 plays a direct role in the proteolytic processing of NCAM2 in hippocampal neurons, cultured hippocampal neurons were treated for 24 h with β-secretase inhibitor IV. Western blot analysis with an antibody against the first Fn repeat of NCAM2-ED (Supplementary Fig. S1) which is located N-terminally to the BACE1-cleavage site (Fig. 1A, [32]) showed that BACE1 inhibition causes an increase in NCAM2 levels in lysates of neurons (Fig. 1B). In the supernatant collected from neuronal cultures, this antibody detected soluble NCAM2, which represents NCAM2-ED, as indicated by molecular weight determination (Fig. 1B). BACE1 inhibition reduced levels of soluble NCAM2 in the culture medium (Fig. 1B) indicating that NCAM2-ED was proteolytically removed from the cell surface by BACE1.

To investigate the effect of BACE1 deficiency on NCAM2 levels, the levels of NCAM2-ED immunoreactivity were analyzed along dendrites of cultured hippocampal neurons co-transfected with GFP together with BACE1 or control siRNAs. Unexpectedly, a ⁓50% reduction in BACE1 levels (Supplementary Fig. S2) did not affect the NCAM2-ED levels along dendrites (Fig. 1C). The intensities of labeling with antibodies against the very C-terminus of the intracellular domain (ID) of NCAM2 (Fig. 1A) were, however, reduced along dendrites (Fig. 1C) and the ratios of NCAM2-ED/NCAM2-ID levels were increased in cultures treated with BACE1 siRNA *vs* control siRNA (Fig. 1C). A similar effect was observed in neurons transfected with BACE2 siRNA (Fig. 1C), with BACE2 being also expressed in hippocampal neurons although at a lower level than BACE1 [47, 48].

To further investigate whether changes in the NCAM2-ED/NCAM2-ID ratio reflect BACE1 activity levels, we used CHO cells, which had been used for the analysis of the cleavage of APP, a well-studied BACE1 substrate [20, 49–51]. Inhibition of BACE1 with β-secretase inhibitor IV did not affect the levels of NCAM2-ED in NCAM2-transfected CHO cells but led to a reduction in the levels of NCAM2-ID and an increase in the ratios of NCAM2-ED/NCAM2-ID labeling intensities (Fig. 1D), as seen in cultured neurons. In contrast, co-expression of BACE1 with human NCAM2 containing an HA tag at the extracellular N-terminus (HA-NCAM2, Fig. 1A) reduced the NCAM2-ED/NCAM2-ID ratio in transfected cells (Supplementary Fig. S3). Similarly, the HA tag/NCAM2-ID ratio was reduced in BACE1-co-trasfected CHO cells (Fig. 1E). This reduction was reflected by a nearly complete loss of the HA tag and NCAM2-ED immunoreactivity in BACE1-co-transfected cells, whereas NCAM2-ID was retained (Fig. 1E, Supplementary Fig. S3). Results indicate that the NCAM2-ED/NCAM2-ID ratio inversely correlates with BACE1 activity.

Altogether, we conclude that BACE1 is involved in the proteolytic processing of NCAM2 in hippocampal neurons.

### A fragment of NCAM2 accumulates in BACE1-containing endosomes

**Fig. 2:**
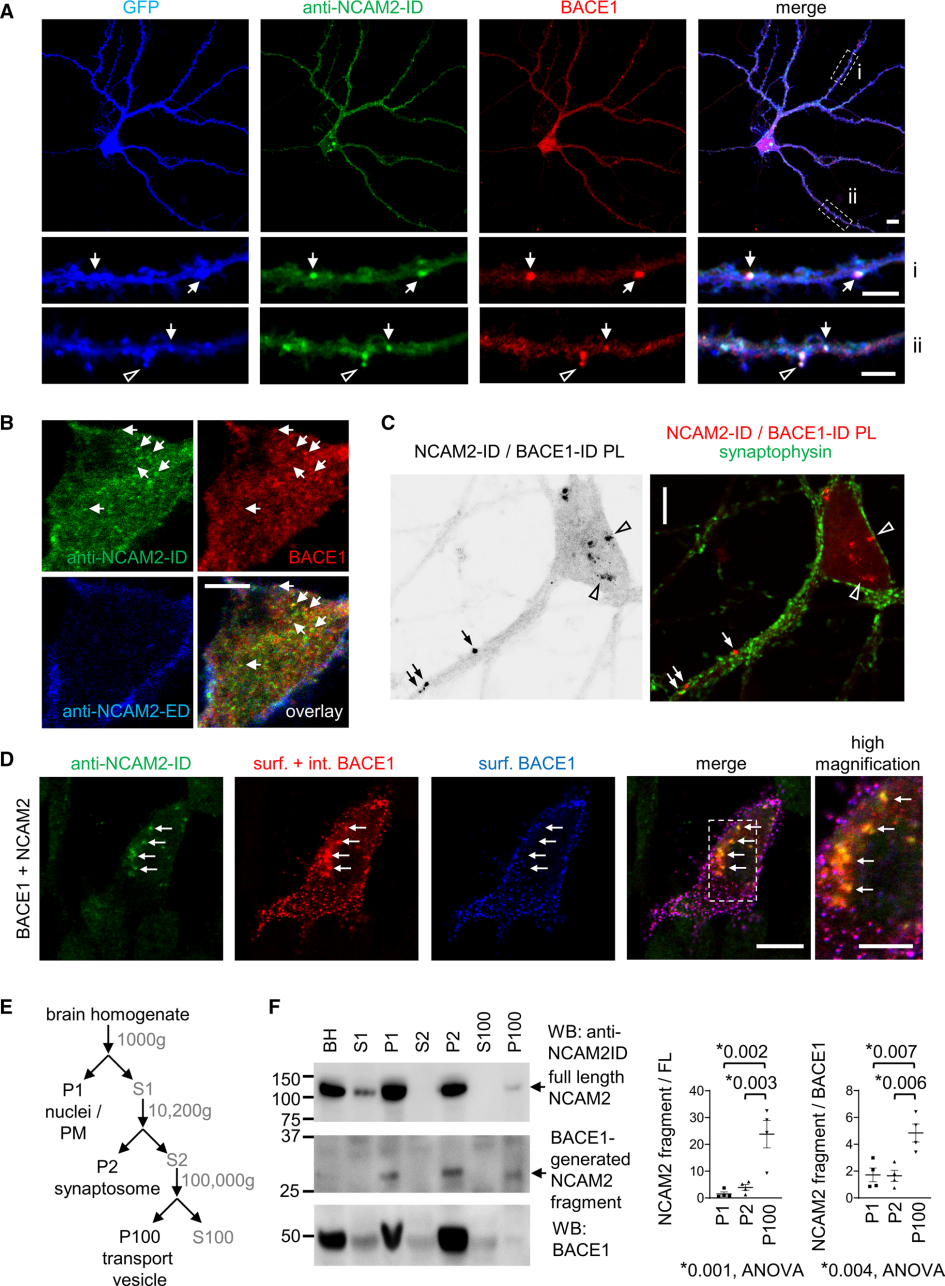
NCAM2-ID and BACE1 co-localize in endosomes. **A** Distribution of NCAM2-ID and BACE1 immunoreactivity in a cultured hippocampal neuron transfected with GFP to visualize its morphology. Note overlapping accumulations of NCAM2-ID and BACE1 in the shaft (arrows) and spine (arrowhead) in the dendrite. Bars, 10 µm (low magnification), 5 µm (high magnification). **B** A confocal slice through a soma showing NCAM2-ED-negative intracellular accumulations of NCAM2-ID immunoreactivity co-localizing with BACE1 (arrows). Bar, 5 µm. **C** The grayscale image shows NCAM2-ID/BACE1-ID proximity ligation (PL) products (black aggregates) in a cultured hippocampal neuron. NCAM2-ID/BACE1-ID PL products are visible in the soma (arrowheads) and in the dendritic shaft (arrows). Co-labeling for synaptophysin shows that the dendritic PL-labeled proteins are detectable in the vicinity of synaptophysin. Bar, 10 µm. **D** Co-localization of internalized BACE1 and NCAM2-ID accumulations (arrows) in a CHO cell co-transfected with BACE1 and NCAM2. Accumulations of internalized BACE1 are seen as red clusters of BACE1 detected after surface membrane permeabilization (surf. + int. BACE1). These clusters do not overlap with clusters of BACE1 detected before permeabilizing membranes (surf. BACE1). Bars, 10 μm (low magnification), 5 µm (high magnification). **E** Scheme of the subcellular fractionation protocol used to produce fractions analyzed in **F**. **F** Western blot analysis of the brain homogenate (BH) and fractions obtained as shown in **E**. Note that BACE1 and the ~ 32 kDa NCAM2 fragment detected with NCAM2-ID antibodies, which was previously described as the cleavage product of BACE1 [32], are present in fraction P100 enriched in transport vesicles. This fragment is not detectable in fraction S100 containing soluble proteins. Graphs show the enrichment of this fragment relative to full-length NCAM2 levels (NCAM2 fragment/FL) and relative to BACE1 levels (NCAM2 fragment/BACE1) in corresponding fractions (mean ± SEM, *n* = 3) normalized to the enrichment in BH set to 1. *p, ANOVA with Tukey’s multiple comparisons test

In cultured hippocampal neurons analyzed by confocal microscopy, accumulations of NCAM2-ID and BACE1 immunoreactivities co-localized in vesicle-like structures in dendritic shafts, occasionally in dendritic spines (Fig. 2A) and in somata (Fig. 2B). Accumulations of NCAM2-ID immunoreactivity co-localizing with BACE1 were often not detectable with NCAM2-ED antibodies (Fig. 2B). Out of 313 clusters of BACE1 identified manually in dendrites, 128 clusters colocalized with a cluster of NCAM2-ID immunoreactivity and only 64 clusters also colocalized with a cluster of NCAM2-ED immunoreactivity (31 neurons from 2 cultures were analyzed).

Proximity ligation (PL) using antibodies against NCAM2-ID and BACE1-ID produced a positive reaction (Fig. 2C). The reaction was not observed when the BACE1 antibodies were omitted (Supplementary Fig. S4). The NCAM2-ID/BACE1-ID ligation products were present along dendrites, occasionally near synaptophysin-positive synaptic boutons, and in somata (Fig. 2C) showing that NCAM2-ID and BACE1-ID are in close proximity (40 nm or less) and may even form a complex containing the two molecules.

To confirm that BACE1 co-localizes with NCAM2-ID in endosomes, CHO cells co-transfected with BACE1 and NCAM2 were incubated live with antibodies against BACE1-ED for 30 min at 37 °C. BACE1-ED antibodies bound to BACE1 remaining at the cell surface were then visualized with fluorochrome-conjugated secondary antibodies applied to live cells on ice. BACE1-ED antibodies internalized together with BACE1 into endosomes were then visualized after fixing and detergent-permeabilizing the cells using secondary antibodies conjugated to another fluorochrome. Co-labeling of cells for NCAM2-ID showed that accumulations of NCAM2-ID immunoreactivity indeed co-localized with clusters of internalized BACE1 (Fig. 2D).

To determine whether NCAM2 co-localizes with BACE1 in transport vesicles of the brain, the transport vesicle enriched fraction (P100), containing vesicles involved in BACE1 transport in neurons [27], was isolated from brain tissue by differential centrifugation [27, 46], by sequentially depleting the mouse brain tissue of nuclei and plasma membranes (fraction P1) [52], synaptosomes (fraction P2) and soluble proteins [46] (Fig. 2E). Western blot analysis of these fractions with NCAM2-ID antibody showed that full-length NCAM2 was enriched in fractions P1 and P2 (Fig. 2F) in accordance with NCAM2 localization at the plasma membrane (Fig. 2B) and synapses [11]. Low levels of full-length NCAM2 were also detected in fraction P100 enriched in transport vesicles (Fig. 2F). The NCAM2-ID antibody also detected a fragment of NCAM2 previously described as a BACE1-generated cleavage product and detectable at ~ 32 kDa [32], which was present at similar levels in fractions P1, P2 and P100 (Fig. 2F). This fragment was not detectable in the soluble protein fraction S100 (Fig. 2F), indicating that it is a membrane-bound NCAM2 fragment. The enrichment of the 32 kDa fragment relative to full-length NCAM2, determined as the ratio of their levels in respective fractions was highly increased in transport vesicles (Fig. 2F). Furthermore, the enrichment of this fragment relative to BACE1, determined as the ratio of the levels of these two proteins was also highly increased in transport vesicles (Fig. 2F).

Altogether, our data indicate that the NCAM2 fragment most likely representing the previously described BACE1-generated cleavage product accumulates in vesicles involved in BACE1 transport.

### NCAM2-ED is not required for the NCAM2-ID/BACE1 complex formation and its targeting to endosomes

**Fig. 3:**
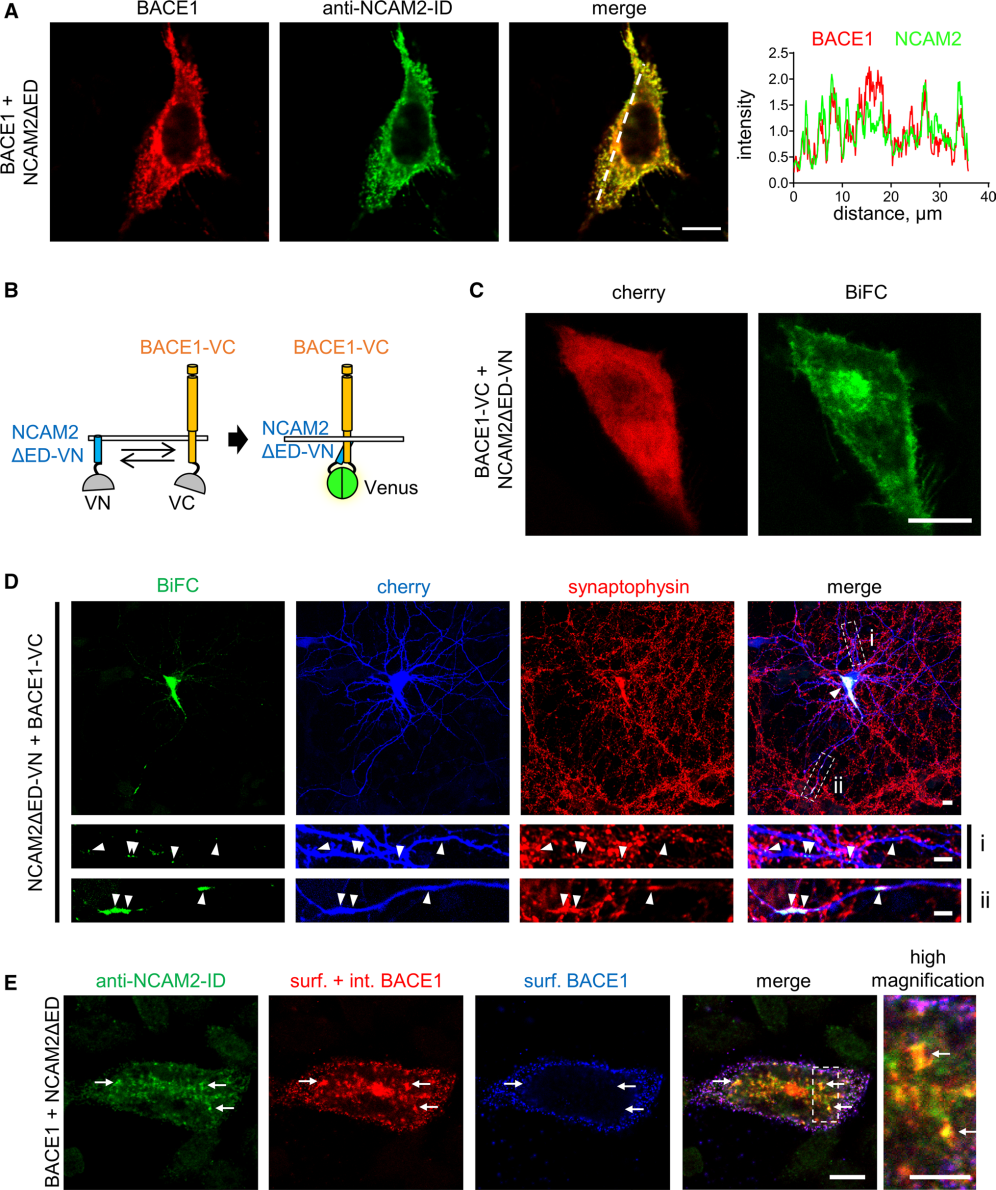
NCAM2-ED is not required for targeting of NCAM2-ID to BACE1. **A** NCAM2-ID and BACE1 distribution in a CHO cell co-transfected with NCAM2ΔED and BACE1. Graph shows the distribution of labeling intensities along the dashed line. Bar, 10 µm. **B** Schematic diagram showing the method of BiFC. Reconstitution of the fluorescent protein Venus from VN and VC fragments fused to the C-termini of NCAM2ΔED and BACE1 results in fluorescence. **C** BiFC signals in CHO cells co-transfected with BACE1-VC and NCAM2ΔED-VN and cherry fluorescent protein. Bar, 20 μm. Note BiFC signals at the plasma membrane and cytoplasm. **D** BiFC staining in a cultured hippocampal neuron co-transfected with BACE1-VC, NCAM2ΔED-VN, and cherry fluorescent protein, and co-labelled for synaptophysin. Arrowheads show BiFC in the soma (low magnification image; bar, 10 µm), dendrites (i) and axon (ii) (high magnification images; bar, 5 µm). Axonal BiFC signals co-localize with synaptophysin accumulations. **E** Co-localization of internalized BACE1 and NCAM2-ID accumulations (arrows) in a CHO cell co-transfected with BACE1 and NCAM2ΔED. Accumulations of internalized BACE1 are seen as red clusters of BACE1 detected after the permeabilization of membranes (surf. + int. BACE1), which do not overlap with clusters of BACE1 detected before permeabilizing membranes (surf. BACE1). Bars, 10 μm (low magnification), 5 µm (high magnification)

We next analyzed whether NCAM2-ED is required for targeting of NCAM2-ID to BACE1. In CHO cells co-transfected with the NCAM2 fragment comprising only the transmembrane and intracellular domains (NCAM2ΔED), NCAM2-ID and BACE1 immunolabeling highly co-localized at the plasma membrane and in vesicular-like structures (Fig. 3A).

Bimolecular fluorescence complementation (BiFC) assay, allowing to visualize proteins located from each other within ⁓ 4.2 nm, i.e. the size of the Venus fluorescent protein [35], was then used to test whether BACE1 and NCAM2ΔED form a complex. In this assay, non-fluorescent N-terminal (VN) and C-terminal (VC) fragments of Venus were fused to the cytosolic C-termini of NCAM2ΔED and BACE1, respectively, and the re-formation of the fluorescent full-length Venus protein from VN and VC tags at sites of proximity between NCAM2 and BACE1 was then analyzed. Co-transfection of CHO cells with NCAM2ΔED-VN and BACE1-VC led to the reconstitution of Venus and generation of BiFC signals at the plasma membrane and in the cytoplasm (Fig. 2C). Formation of NCAM2ΔED-VN/BACE1-VC BiFC was inhibited in a dominant-negative manner in cells co-transfected with BACE1 without the VC tag (Supplementary Fig. S5), indicating that BiFC depended on the interaction between NCAM2ΔED and BACE1 and that this interaction was not caused by the non-specific association of the VN and VC tags.

Co-transfection of cultured hippocampal neurons with NCAM2ΔED-VN and BACE1-VC also resulted in BiFC signals detected along dendrites, in somata and synaptophysin-positive structures in axons (Fig. 3D) indicating that NCAM2ΔED associates with BACE1 and that the NCAM2-ED is not required for this association.

In CHO cells, NCAM2ΔED also co-localized with clusters of internalized BACE1 detected by incubating live cells with antibodies against BACE1-ED for 30 min at 37 °C (Fig. 3E).

The combined observations allow us to conclude that the transmembrane and intracellular domains of NCAM2 are sufficient for the interaction with BACE1, which occurs independently of NCAM2-ED.

### The association of NCAM2 and BACE1 precedes endocytosis

**Fig. 4:**
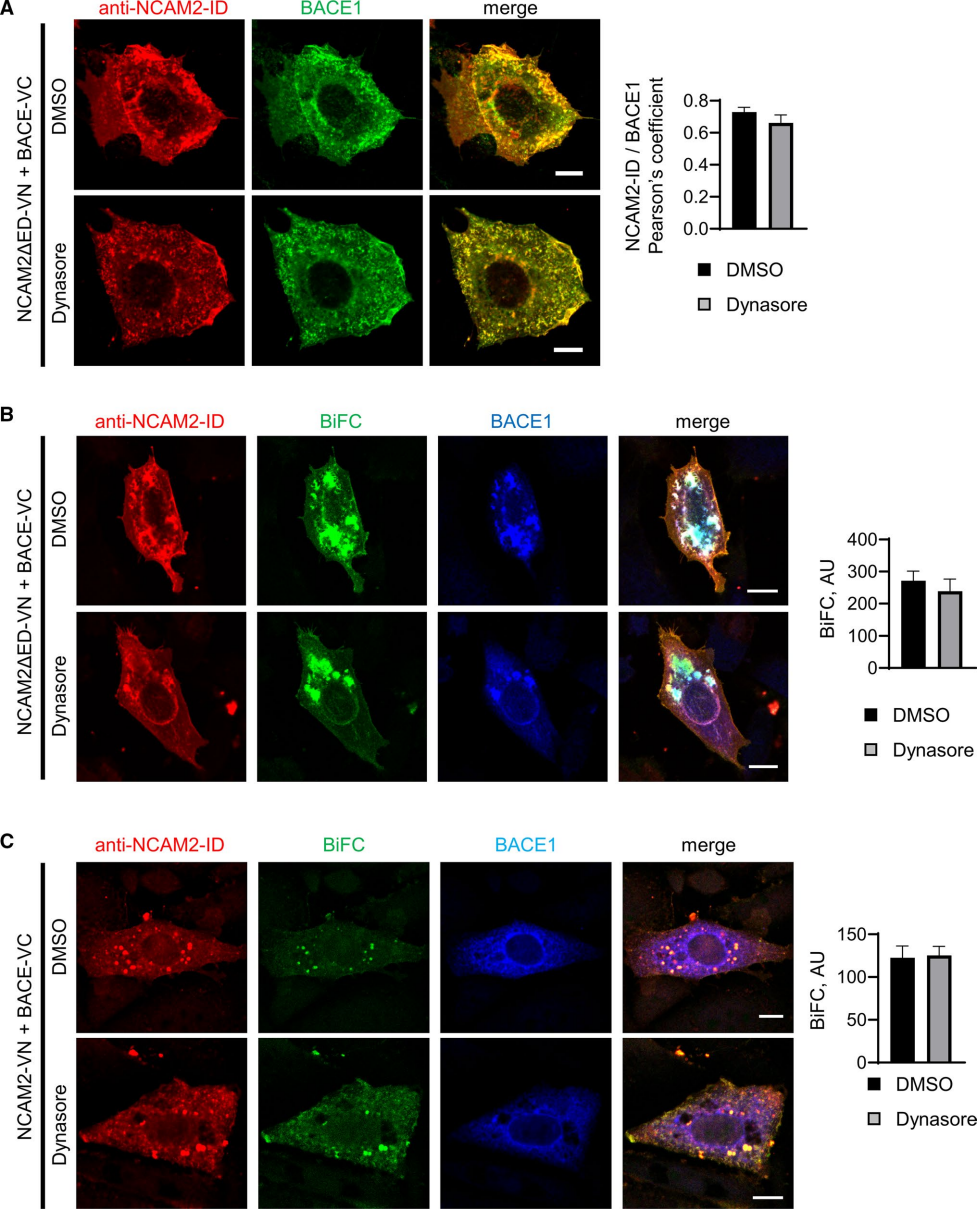
Inhibition of endocytosis does not reduce the interaction of NCAM2 and BACE1. **A** BACE1 and NCAM2-ID in CHO cells co-transfected with BACE1 and NCAM2ΔED and treated with dynasore or vehicle (0.1% DMSO). Graph shows mean + SEM and Pearson’s coefficient of BACE1 and NCAM2-ID co-localization (*n* > 20 cells). **B, C** BiFC in CHO cells co-transfected with BACE1-VC and NCAM2ΔED-VN (**B**) or NCAM2-VN (**C**). Cells were either treated with dynasore or vehicle (0.1% DMSO), and co-labelled for NCAM2-ID and BACE1. Graph shows mean + SEM BiFC fluorescence intensities. *n* = 50 (DMSO) and 22 (dynasore) cells in **B**, *n* = 13 (DMSO) and 13 (dynasore) cells in **C**. Bars = 20 μm

BACE1 is associated with its well-characterized substrate APP in endosomes and their approximation is blocked by the endocytosis inhibitor dynasore [27]. In contrast, the NCAM2ΔED/BACE1 BiFC complexes were found at the plasma membrane (Fig. 3C) suggesting that their formation precedes endocytosis. Indeed, dynasore did not reduce the co-localization of NCAM2ΔED and BACE1 in transfected CHO cells (Fig. 4A) and did not affect the BiFC signal levels produced in CHO cells co-transfected with NCAM2ΔED-VN/BACE1-VC (Fig. 4B). Similarly, co-transfection of CHO cells with the VN-tagged full-length NCAM2 (NCAM2-VN) and BACE1-VC also led to the reconstitution of Venus and generation of BiFC signals, which were not affected by dynasore (Fig. 4C). Our results suggest that the association of NCAM2 with BACE1 precedes endocytosis and might occur at the cell surface.

### NCAM2ΔED co-accumulates with BACE1 in Rab11-positive recycling endosomes

**Fig. 5:**
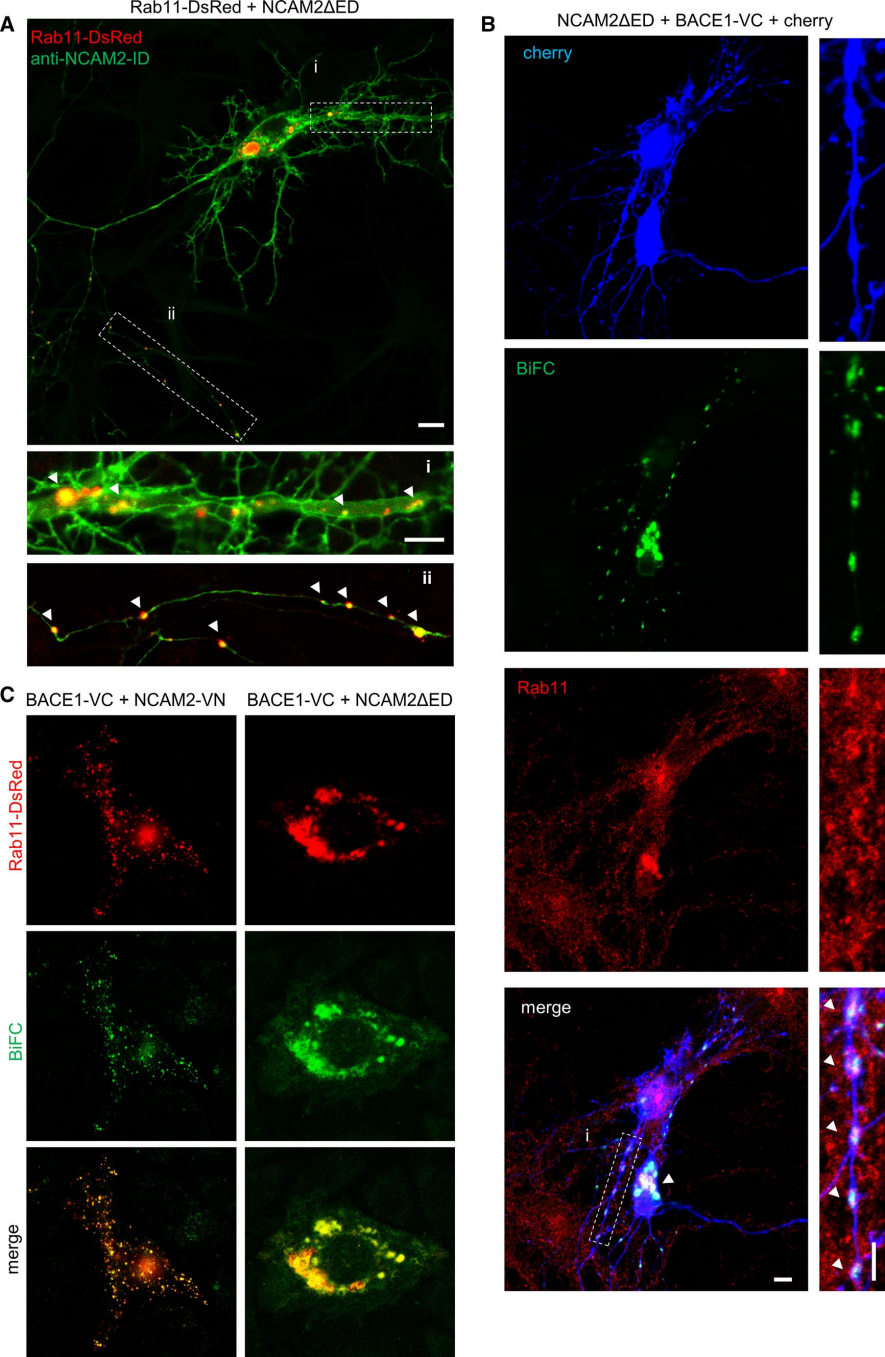
NCAM2ΔED associates with BACE1 in Rab11-positive recycling endosomes. **A** NCAM2-ID labeling in a cultured hippocampal neuron co-transfected with Rab11-DsRed and NCAM2ΔED. High magnification images show examples of NCAM2-ID accumulations in Rab11-DsRed positive endosomes (arrowheads) in dendrites (i) and axon (ii). Bar = 10 µm (low magnification), 5 µm (high magnification). **B** BiFC in cultured hippocampal neurons co-transfected with cherry, NCAM2ΔED-VN and BACE1-VC and labelled for Rab11. Arrowheads show examples of BiFC co-localized with Rab11 in the soma (low magnification image) and along dendrites (high magnification image). Bar, 10 µm (low magnification), 5 µm (high magnification). **C** BiFC in CHO cells co-transfected with Rab11-DsRed, BACE1-VC and NCAM2-VN or NCAM2ΔED-VN. Bar, 10 µm

BACE1 endocytosed from the dendritic cell surface is incorporated into early or recycling endosomes [20, 25, 27]. The BACE1-containing early endosomes are mostly stationary in dendrites [27], whereas the recycling Rab11-positive endosomes are involved in the transport of BACE1 from the dendrites and its trafficking to axons and axonal terminals [24]. Presence of NCAM2ΔED-VN/BACE1-VC BiFC in axons (Fig. 3D) suggested that NCAM2ΔED co-accumulates with BACE1 in the recycling Rab11-positive endosomes. Indeed, in cultured hippocampal neurons co-transfected with NCAM2ΔED and Rab11-DsRed, NCAM2-ID immunoreactivity accumulated in Rab11-positive endosomes in both dendrites and axons identified morphologically (Fig. 5A). BiFC complexes formed by NCAM2ΔED-VN and BACE1-VC along dendrites, axons, and in somata were also found in Rab11-positive endosomes as detected with antibodies against endogenous Rab11 (Fig. 5B). In CHO cells co-transfected with BACE1, Rab11-DsRed and NCAM2 or NCAM2ΔED, NCAM2-ID labeling also highly co-localized with BACE1 in Rab11-DsRed-positive recycling endosomes (Fig. 5C). In summary, our data indicates that NCAM2ΔED co-accumulates with BACE1 in Rab11-positive recycling endosomes.

### NCAM2 promotes targeting of BACE1 to Rab11-positive recycling endosomes

**Fig. 6:**
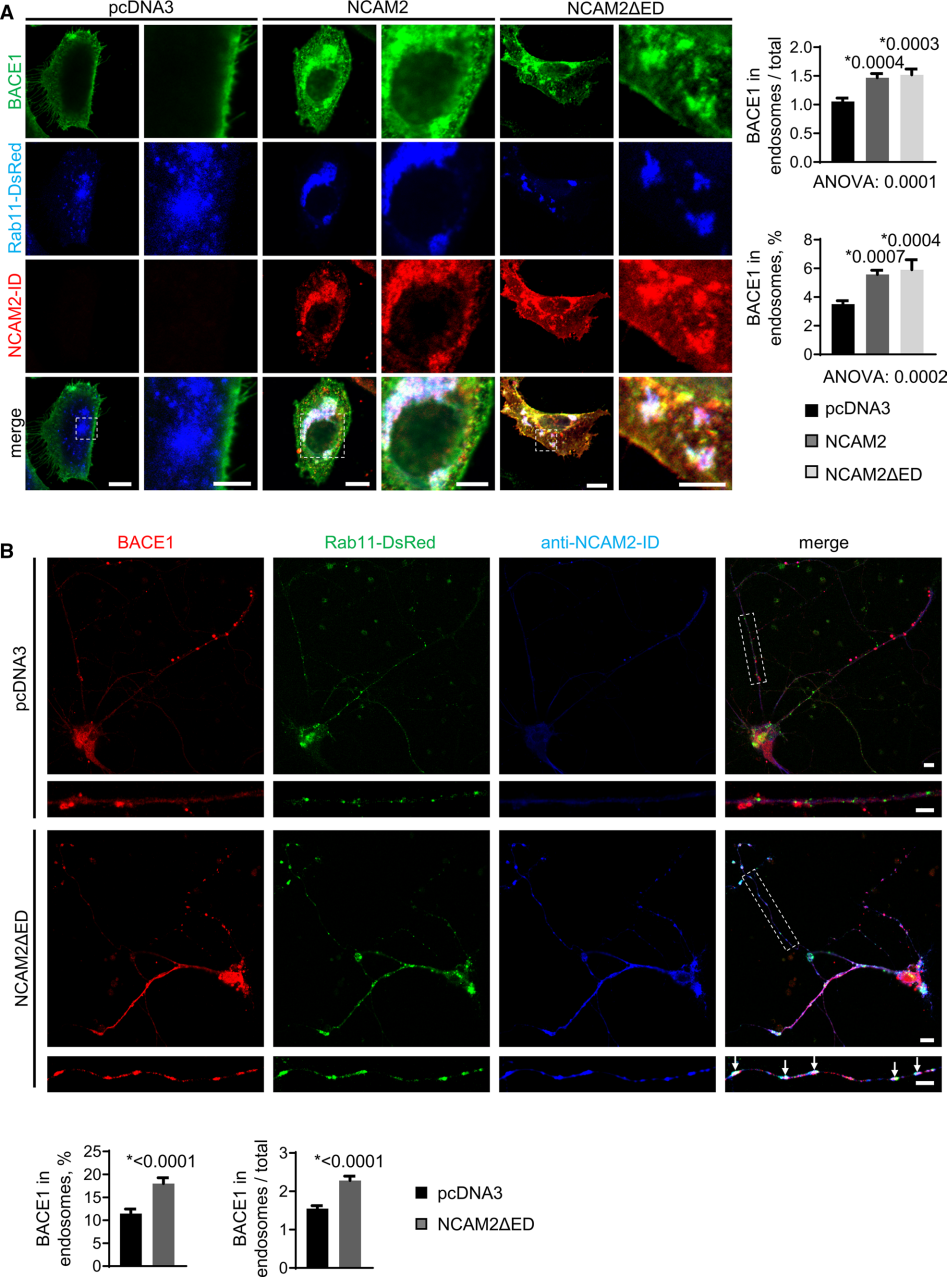
NCAM2ΔED promotes targeting of BACE1 to Rab11-positive endosomes. **A** NCAM2-ID and BACE1 labeling in CHO cells co-transfected with BACE1, Rab11-DsRed and full-length NCAM2, NCAM2ΔED or empty pcDNA3 vector. Graphs show mean + SEM of BACE1 levels in endosomes relative to the total BACE1 levels and percentages of BACE1 in endosomes (*n* > 36). *p, one-way ANOVA and Dunnett’s multiple comparisons test, compared to pcDNA3. Bars, 10 µm (low magnification), 5 µm (high magnification). **B** BACE1 levels in cultured hippocampal neurons co-transfected with Rab11-DsRed and NCAM2ΔED or empty pcDNA3 vector. Note higher co-localization of BACE1 with Rab11-positive endosomes in NCAM2ΔED overexpressing neurons (arrows). Graphs show mean + SEM of levels of BACE1 in Rab11-positive endosomes relative to total BACE1 levels and percentages of BACE1 in Rab11-positive endosomes (*n* = 78). *p, Mann–Whitney test. Bar, 10 µm (low magnification), 5 µm (high magnification)

The observation of the close association of NCAM2ΔED with BACE1 prompted us to analyze whether NCAM2 regulates the targeting of BACE1 to recycling endosomes. First, we compared the levels of BACE1 in Rab11-positive endosomes in CHO cells co-transfected with BACE1 and either the control pcDNA3 vector or NCAM2. In NCAM2-transfected cells, accumulations of NCAM2-ID immunoreactivity co-localized with BACE1 in Rab11-positive endosomes (Fig. 6A). The enrichment of BACE1 in individual Rab11-positive recycling endosomes relative to the total BACE1 levels in the cell and the proportion of BACE1 in these organelles were increased in NCAM2-transfected *vs* control vector-transfected cells (Fig. 6A). A similar increase was found in CHO cells co-transfected with NCAM2ΔED instead of NCAM2 (Fig. 6A), indicating that the targeting of BACE1 to Rab11-positive endosomes is mediated by amino acid sequences present in NCAM2ΔED independently of the extracellular domain of NCAM2. From the endosomal compartment, BACE1 can be transported to the trans-Golgi [22] or to lysosomes for degradation [21]. The levels of BACE1 were similar in Golgi organelles (Supplementary Fig. S6) and only slightly increased in lysosomes (Supplementary Fig. S7) in NCAM2- or NCAM2ΔED-transfected *vs* control vector-transfected CHO cells. The levels of BACE1 in the endoplasmic reticulum were not changed in NCAM2- or NCAM2ΔED-transfected CHO cells (Supplementary Fig. S8). Overexpression of NCAM2ΔED in cultured hippocampal neurons also enriched BACE1 in individual Rab11-positive recycling endosomes and increased the proportion of BACE1 in these organelles (Fig. 6B).

Altogether, our data indicate that NCAM2 targets BACE1 to the recycling endosomes, does not increase its targeting to other organelles and does not change its export from the endoplasmic reticulum.

### Rab11 levels are downregulated in NCAM2-deficient mice

**Fig. 7:**
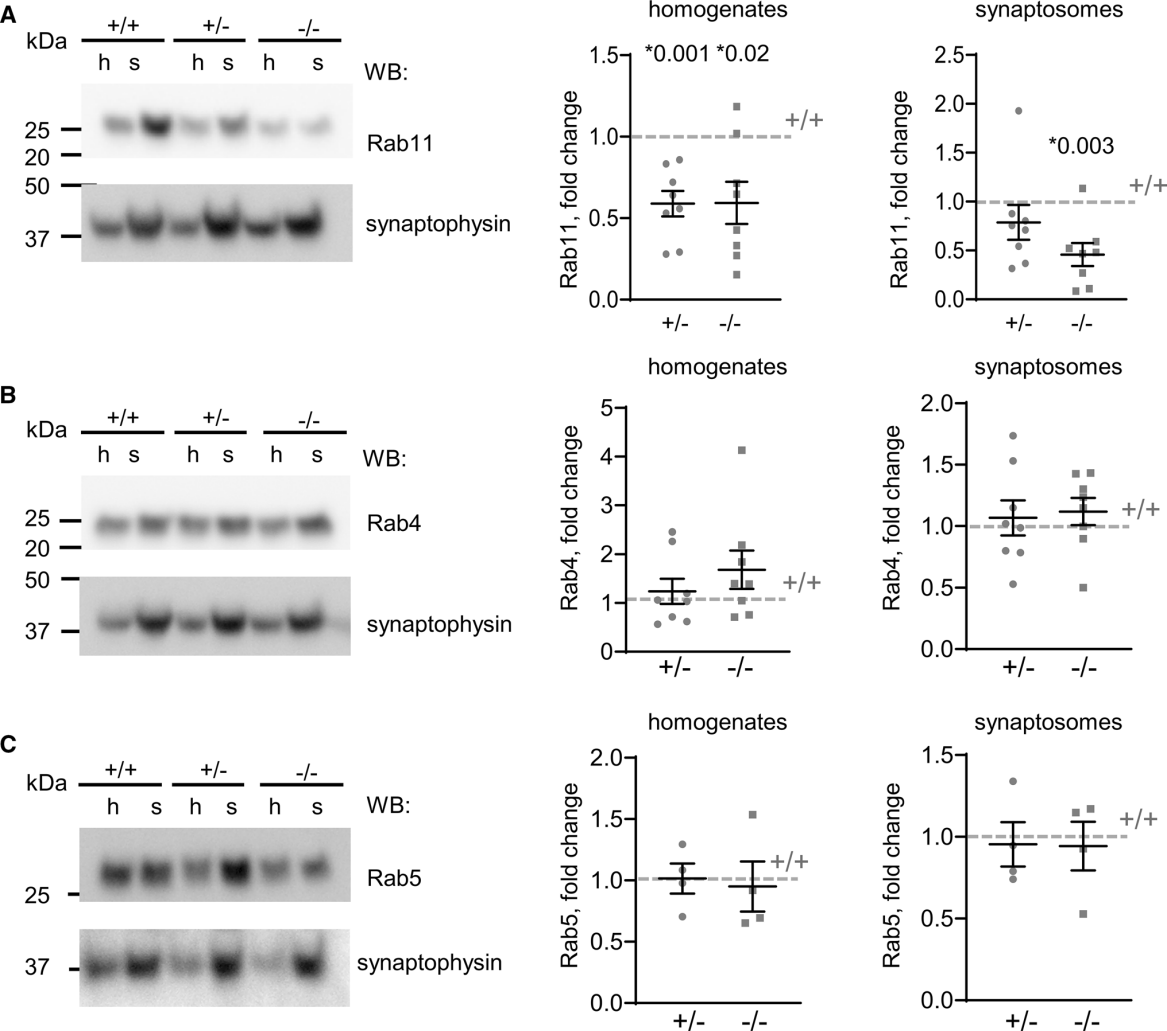
Rab11 expression is downregulated in brains of NCAM2−/− mice. **A**–**C** Levels of Rab11 (**A**), Rab4 (**B**), and Rab5 (**C**) in brain homogenates (h) and synaptosomes (s) from NCAM2 + / + , NCAM2+/− and NCAM2−/− mice as indicated by Western blot analysis. Graphs show mean ± SEM changes (in fold) in homogenates and synaptosomes of NCAM2+/− and NCAM2−/− mice relative to NCAM2 + / + levels (*n* = 8). *p, one sample *t* test, compared to + / +

Rab11-positive slowly recycling endosomes are involved in regulating the turnover of proteins in the post-synaptic density [53, 54], where NCAM2 is present [11]. Western blot analysis demonstrated that Rab11 is enriched in synaptosomes from NCAM2 + / + brains (Fig. 7A). The levels of Rab11 were, however, strongly reduced in NCAM2−/− brain homogenates and synaptosomes and were also reduced in NCAM2+/− mice (Fig. 7A). In contrast, the levels of Rab4 and Rab5, which localize to the rapid recycling and early endosomes, respectively, were not affected (Fig. 7B,C). Altogether, these data indicate that NCAM2 specifically regulates Rab11-positive recycling endosomes.

### NCAM2 deficiency causes accumulation of BACE1 at the dendritic cell surface

**Fig. 8:**
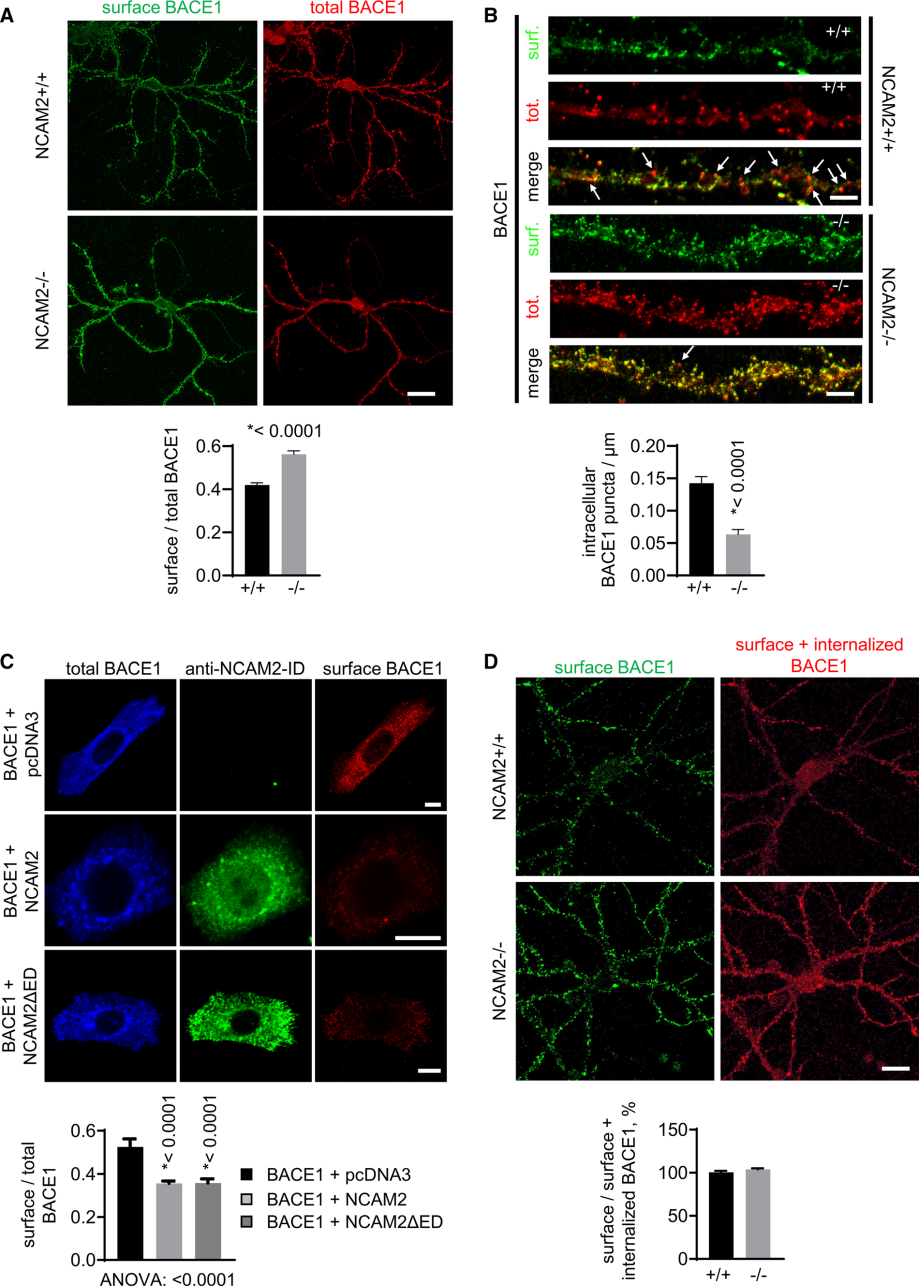
NCAM2 reduces cell surface levels of BACE1. **A, B** Cell surface and total BACE1 visualized in cultured NCAM2 + / + and NCAM2−/− hippocampal neurons (**A**). Higher magnification of dendrites is shown in **B**. Note higher cell surface BACE1 levels in NCAM2−/− neurons (**A, B**), and higher numbers of intracellular BACE1 accumulations (arrows) in dendrites of NCAM2 + / + neurons (**B**). Graphs show mean + SEM ratios of cell surface and total BACE1 labeling intensities along dendrites (**A**) and densities of intracellular BACE1 accumulations (**B**). *p, unpaired *t* test (*n* = 120). Bar, 20 µm (**A**), 5 µm (**B**). **C** Cell surface and total BACE1 visualized in CHO cells co-transfected with BACE1 and pcDNA3 (control), full-length NCAM2 or NCAM2ΔED and co-labelled for NCAM2-ID. Note reduced cell surface BACE1 levels in NCAM2 and NCAM2ΔED co-transfected cells. Graph shows mean ± SEM ratios of cell surface and total BACE1 levels. *p, one-way ANOVA and Dunnett’s multiple comparisons test, compared to pcDNA3 (*n* = 37 (pcDNA3), 34 (NCAM2), 46 (NCAM2ΔED)). **D** Labeling of the cell surface bound pool of BACE1 antibody (surface BACE1) and combined cell surface bound and internalized pool of BACE1 antibody (surface + internalized BACE1) in cultured NCAM2 + / + and NCAM2−/− hippocampal neurons incubated with antibodies against the extracellular domain of BACE1 for 30 min at 37 °C. Graph shows mean + SEM ratios of the cell surface and combined pools (*n* = 180 (+ / +), 193 (−/−))

BACE1 is targeted to the endosomal compartment from the cell surface and can be recycled back to the cell surface from endosomes [18]. To determine how changes in endosomal targeting affect the cell surface localization of BACE1, BACE1 at the neuronal cell surface was visualized by labeling live NCAM2 + / + and NCAM2−/− hippocampal neurons with antibodies against BACE1-ED applied on ice. We found that cell surface BACE1 was predominantly detected in morphologically identified dendrites (Fig. 8A). The ratio of cell surface BACE1 levels to total BACE1 levels detected by co-labeling neurons with BACE1 antibodies after detergent permeabilization was higher in dendrites of NCAM2−/− than NCAM2 + / + neurons (Fig. 8A), indicating that NCAM2 deficiency causes accumulation of BACE1 at the dendritic cell surface. An increase of BACE1 levels at the cell surface was accompanied by a reduction in numbers of intracellular clusters of BACE1 in NCAM2−/− neurons (Fig. 8B), further suggesting a redistribution of BACE1 to the dendritic cell surface.

In CHO cells transfected with BACE1-FLAG, co-transfection with NCAM2 also led to a reduction in the levels of BACE1 at the cell surface relative to the total levels of BACE1 (Fig. 8C). A similar effect was found in CHO cells co-transfected with NCAM2ΔED (Fig. 8C), indicating that this NCAM2 fragment is sufficient to reduce BACE1 levels at the cell surface.

To analyze whether the accumulation of BACE1 at the cell surface of NCAM2−/− neurons is caused by changes in endocytosis rates, live hippocampal neurons of NCAM2 + / + and NCAM2−/− mice were incubated with antibodies against the extracellular domain of BACE1 for 30 min at 37 °C to allow antibody internalization via endocytosis. Neurons were then gently fixed and cell surface and total pools of BACE1 antibody were visualized with secondary antibodies of different colors applied to neurons before and after permeabilization. Analysis of the confocal images of neurons showed that, while the cell surface levels of BACE1/antibody complexes were higher in NCAM2−/− neurons, the total pool of the BACE1/antibody complexes comprising the cell surface and endocytosed pools was also increased, and the ratio of the cell surface and total levels of BACE1/antibody complexes was similar in NCAM2 + / + and NCAM2−/− neurons (Fig. 8D) indicating similar endocytosis rates.

Our results thus indicate that reduced targeting of BACE1 to Rab11-positive endosomes in NCAM2−/− neurons leads to the accumulation of BACE1 at the cell surface most likely caused by increased recycling of BACE1 from the endosomal compartment to the cell surface.

### BACE1 shedding is increased, and its activity is reduced in NCAM2−/− brains

**Fig. 9:**
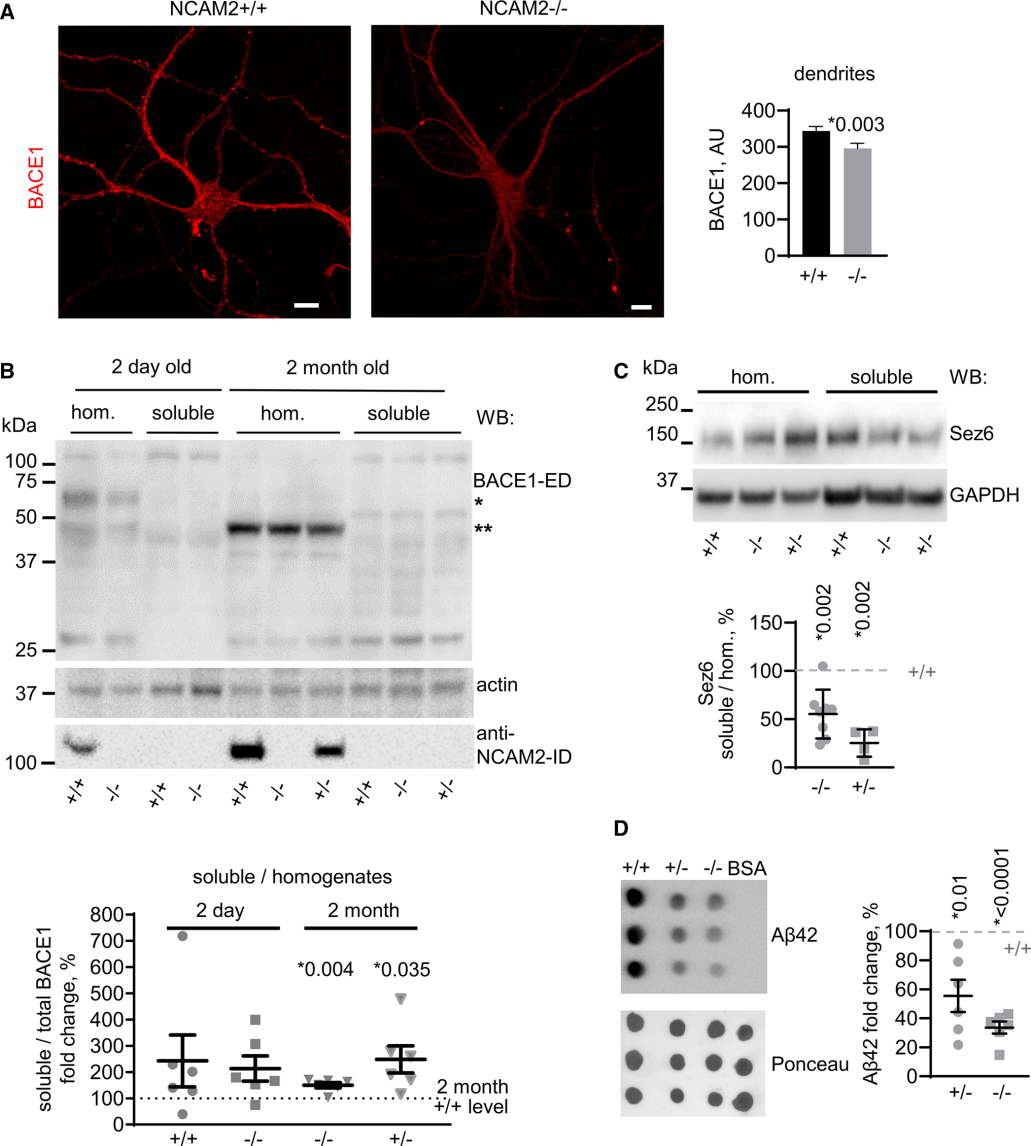
Shedding of BACE1 from the cell surface is increased and BACE1 activity is reduced in NCAM2−/− mice. **A** Total BACE1 visualized in cultured NCAM2 + / + and NCAM2−/− hippocampal neurons. Graph shows mean + SEM labeling intensities of BACE1 in dendrites of neurons (*n* = 60). *p, Mann–Whitney test. Bar, 10 µm. **B** Total BACE1 levels in brain homogenates (hom.) and soluble BACE1 levels in soluble protein fractions (soluble) from 2-day-old and 2-month-old NCAM2 + / + , NCAM2+/− and NCAM2−/− mice as detected by Western blot analysis with BACE1-ED antibodies. Actin served as a loading control. NCAM2-ID labeling shows higher levels of NCAM2 in the adult brain. Glycosylated (*) and non-glycosylated (**) BACE1 [56] are enriched in brain homogenates from 2-day-old and 2-month-old mice, respectively. Bands corresponding to soluble BACE1-ED in the soluble protein fraction are ~ 6 kDa smaller than full-length BACE1. A BACE1-immunoreactive band at ~ 110 kDa most likely represents dimers of BACE1-ED [86]. Lower molecular weight degradation products of BACE1 are also detected in the soluble protein fraction. Graph shows mean + SEM change (in fold) of the ratio of soluble *vs* total BACE1 levels relative to 2-month-old NCAM2 + / + brains set to 100% (*n* = 6). *p, one sample *t* test. **C** Total levels of Sez6 levels in brain homogenates and soluble Sez6 levels in soluble protein fractions from 2-month-old NCAM2 + / + , NCAM2+/− and NCAM2−/− mice as detected by Western blot analysis. GAPDH served as a loading control. Graph shows mean ± SEM change (in fold) in the ratio of soluble and total Sez6 levels relative to + / + brains set to 100% (*n* = 8 (−/−), 4 (+/−)). *p, one sample *t* test, compared to + / + . **D** Dot blot analysis of Aβ42 levels in brain homogenates from 5–6 months-old NCAM2 + / + , NCAM2+/− and NCAM2−/− mice. Samples from each animal were analyzed in triplicates. Graph shows mean ± SEM change (in fold) in Aβ42 levels relative to + / + brains set to 100% (*n* = 6 mice per group). *p, one sample *t*-test, compared to + / +

While the cell surface levels of BACE1 were increased in dendrites of NCAM2−/− neurons (Fig. 8A), the total pool of BACE1 detected by immunolabeling of detergent-permeabilized cultured hippocampal neurons with antibodies against BACE1 was reduced in dendrites of NCAM2−/− neurons (Fig. 9A).

Since BACE1-ED can be shed from the cell surface via proteolytic cleavage of BACE1 near the plasma membrane [29, 30], we asked whether the accumulation of BACE1 at the cell surface of NCAM2−/− dendrites causes increased shedding of BACE1-ED. Western blot analysis with antibodies against BACE1-ED indicated that the ratio of BACE1 cleavage products in the soluble protein fraction versus BACE1 levels in total brain homogenates was increased in 2-month-old NCAM2−/− and NCAM2+/− mice (Fig. 9B), indicating an increase in BACE1 shedding. The levels of the transmembrane NCAM2 isoform increase with age (6.3 ± 1.5-fold higher (*n*  = 3) in the brains of 2-month-old versus 2-day-old NCAM2 + / + mice (Fig. 9B)). The levels of BACE1 shedding were similar in brain homogenates of 2-day-old NCAM2 + / + and NCAM2−/− mice containing predominantly the highly glycosylated form of BACE1 as detected by its mass ~ 65 kDa [55, 56], further indicating that shedding of BACE1 is inversely correlated with NCAM2 levels.

BACE1 is most active in the acidic environment of endosomes and trans-Golgi network, rather than at the cell surface [57]. Furthermore, soluble BACE1 is unable to proteolytically process its substrates [31]. To determine whether an increase in BACE1 cell surface localization and shedding affects the activity of the enzyme, shedding of Sez6, which is shed exclusively via the BACE1-mediated cleavage [58, 59] was analyzed and found to be strongly reduced in the brains of 2-month-old NCAM2−/− mice (Fig. 9C). Since BACE1 is a rate-limiting enzyme in the pathway leading to Aβ generation, the levels of Aβ42 were determined and found to be also strongly reduced in brain homogenates from NCAM2−/− vs NCAM2 + / + mice, and an intermediate effect was found in NCAM2+/− mice (Fig. 9D). We interpret these findings to show that the loss of NCAM2 leads to reduced activity of BACE1.

Our observations thus indicate that NCAM2 deficiency causes a reduction in BACE1 activity, most likely due to its increased shedding from the cell surface.

## Discussion

Substrate cleavage by BACE1 is increasingly appreciated to play important roles in modifying or activating the function of substrates [60–63]. Whether the cleavage products generated by BACE1 regulate BACE1 is however unknown. In our work, we confirm a previous study showing that NCAM2 is a BACE1 substrate [32]. Our data showing that the inhibition of BACE1 in hippocampal neurons results in the reduced release of soluble NCAM2 with the molecular weight corresponding to the size of NCAM2 ectodomain indicates that BACE1 cleaves NCAM2 in these neurons at the membrane-proximal extracellular site as reported previously [32]. Furthermore, we demonstrate that the BACE1-generated NCAM2 fragment and the recombinant fragment of NCAM2 comprising only the transmembrane and intracellular domains associate with and regulate the trafficking and activity of BACE1. These results suggest a scenario in which BACE1 interacts with NCAM2 and cleaves it, with the transmembrane product of this cleavage remaining associated with BACE1 and regulating its activity.

Several lines of evidence indicate that BACE1-mediated NCAM2 cleavage precedes the cleavage of other BACE1 substrates. Delivery of BACE1 to endosomes is required for the interaction with and processing of APP by BACE1 [27]. In contrast, we demonstrate that inhibition of endocytosis does not reduce the interaction of NCAM2 with BACE1. The cleavage of Sez6 by BACE1 most likely also occurs in endosomes since the levels of Sez6 are increased in the brains of Niemann Pick C1 intracellular cholesterol transporter (NPC1) knockout mice, where BACE1 and Sez6 accumulate in recycling endosomes [64]. The cleavage of Sez6 is reduced in brains of NCAM2 deficient mice, indicating that it depends on NCAM2.

BACE1 is found in many different organelle preparations, including the endoplasmic reticulum, Golgi apparatus, cell surface plasma membrane, lysosomes and several types of endosomes [18, 56, 65, 66]. BACE1 activity is higher in acidic environments [67], including the Golgi apparatus [31]. We cannot exclude that BACE1 interacts with NCAM2 and cleaves it early in the biosynthetic pathway since other CAMs are known to interact with cargo proteins and regulate their transport within the biosynthetic pathway [68]. However, this proposition is unlikely since high levels of NCAM2 in the plasma membrane of neurons indicate that the full-length NCAM2 protein reaches the cell surface and therefore does not meet with and is not cleaved by BACE1 on its way to the cell surface. It is however possible that NCAM2 is cleaved by BACE1 at the cell surface, and the suboptimal non-acidic extracellular environment precludes its excessive cleavage by BACE1.

In dendrites, a large pool of BACE1 is detected in the early and recycling endosomes [24, 25, 27]. In non-neuronal cells, cargo from early endosomes can recycle directly to the cell surface via a Rab4-mediated route or can be targeted to the cell surface via a Rab11-mediated recycling route via the perinuclear compartment [69]. Substantial evidence indicates that in neurons Rab11-positive endosomes are involved in the targeting of BACE1 from dendrites to axons through the soma resulting in a concomitant reduction in the dendritic pool of BACE1 available for recycling [24, 25, 70]. Overexpression of the NCAM2 fragment consisting of the transmembrane and intracellular domains of NCAM2 in CHO cells or cultured neurons leads to an increase in BACE1 levels in Rab11-positive endosomes, indicating that the BACE1-generated NCAM2 cleavage product can target BACE1 to Rab11-positive recycling endosomes. An inhibition of the Rab11-dependent transport of BACE1 is also suggested by increased levels of dendritic cell surface BACE1 in NCAM2−/− neurons. This increase cannot be explained by altered endocytosis, since endocytosis rates are similar in NCAM2−/− and NCAM2 + / + neurons, but rather indicates a larger pool of BACE1 to be available for dendritic recycling. In agreement, a similar increase in the cell surface BACE1 levels was found when the sorting of BACE1 from early to recycling endosomes was blocked by the S498A mutation leading to BACE1 accumulation in early endosomes [66].

The proteins destined for different types of endosomes are sorted at the plasma membrane before endocytosis [71, 72]. Our results suggest that NCAM2 associates with BACE1 at the plasma membrane and mediates molecular sorting that leads to the transport of BACE1 to Rab11-positive endosomes. It may also be possible that NCAM2 promotes the transport of BACE1 from early endosomes to Rab11-positive recycling endosomes. Since Rab11-positive recycling endosomes also deliver cargo to the dendritic surface [53, 54], NCAM2 could promote the accumulation of BACE1 in Rab11-positive endosomes by inhibiting the fusion of these endosomes with the dendritic cell surface, thus retaining BACE1 in these endosomes. Finally, NCAM2 can target BACE1 from the plasma membrane directly to Rab11-positive recycling endosomes, thereby bypassing the passage through early endosomes. Recycling endosomes fuse with the dendritic plasma membrane in two modes, either via “full fusion”, which releases all cargo to the plasma membrane, or “display fusion”, in which only a small pore connects the endosome with the plasma membrane, while soluble proteins are released via the pore, with the transmembrane molecules being retained in the endosome [73, 74]. Experiments in non-neuronal cells indicate that transmembrane proteins can be targeted to the recycling endosomes not only via early endosomes but also independently of the early endosomes [75] possibly by recruitment from the plasma membrane to the recycling endosomes after fusion of the endosome with the plasma membrane. Interestingly, full fusion of recycling endosomes with the cell surface is promoted by signaling through L-type voltage-gated calcium channels [73], which are activated by NCAM2 [6, 9]. NCAM2 may therefore act to bias BACE1 transport via a route which bypasses early endosomes and preferentially targets BACE1 to recycling endosomes from the plasma membrane. Future work should distinguish these possibilities.

We demonstrate that NCAM2 deficiency causes increased cell surface accumulation of BACE1, which correlates with higher shedding of BACE1 in adult NCAM2 deficient mice. The shedding of the BACE1 ectodomain is mediated by the metalloproteinase ADAM10, which functions at the neuronal cell surface [30, 76]. We, therefore, propose that recycling of BACE1 to the dendritic cell surface leads to an increase in the cell surface pool of BACE1 in NCAM2−/− neurons, thereby likely facilitating the ADAM10-dependent shedding of BACE1. Whether changes in the BACE1 shedding and inactivation are coupled to changes in NCAM2 expression levels in the broad spectrum of neuronal functions will be interesting to investigate.

BACE1 activity is increased in the brains of individuals with Alzheimer’s disease [77, 78] and Down syndrome [79]. The levels of NCAM2 are also increased in brains of individuals with AD [11]. NCAM2 is encoded by a gene on chromosome 21 and is also overexpressed in individuals with Down’s syndrome [15]. The results of our present study indicate that an increase in NCAM2 levels can lead to enhanced BACE1 activity. Enhanced processing of BACE1 substrates via increased targeting of BACE1 to Rab11-positive endosomes can also explain a functional link between NCAM2 and Alzheimer’s disease as suggested by studies showing that single nucleotide polymorphisms in the *NCAM2* gene are associated with increased risk of late-onset AD [16] and increased levels of amyloid protein β in the cerebrospinal fluid [17].

NCAM2 deficiency causes neurodevelopmental disorders in humans via mechanisms that remain poorly understood. The current results suggest that, at least partially, abnormalities in brain development and function caused by NCAM2 deficiency can be linked to the reduced function of BACE1, which has been suggested to be associated with changes in synaptic plasticity [80], memory deficits [48, 81], spontaneous seizures [82], defective myelination [83] and guidance of axons [84, 85]. We propose that NCAM2-mediated pathways of BACE1 expression are interesting to investigate as therapeutic targets for treating conditions associated with NCAM2 deficiency or overexpression in humans.

## Supplementary Information

Below is the link to the electronic supplementary material.

Supplementary file1 (PDF 5025 KB)

## Data Availability

The datasets generated during and/or analyzed during the current study are available from the corresponding author upon reasonable request.

## Abbreviations

Aβ: Amyloid β
AD: Alzheimer’s disease
ANOVA: Analysis of variance
APP: Amyloid precursor protein
BACE1: Beta-site amyloid precursor protein cleaving enzyme 1
BACE1-VC: VC-tagged BACE1
BiFC: Bimolecular fluorescence complementation
BSA: Bovine serum albumin
CHO cells: Chinese hamster ovary cells
DMSO: Dimethyl sulfoxide
DNA: Deoxyribonucleic acid
ED: Extracellular domain
EDTA: Ethylenediaminetetraacetic acid
EGTA: Ethylene glycol-bis(β-aminoethyl ether)-*N*,*N*,*N*′,*N*′-tetraacetic acid)
EYFP: Enhanced yellow fluorescent protein
FGF-2: Fibroblast growth factor-2
Fn: Fibronectin type III homologous repeat
GAPDH: Glyceraldehyde 3-phosphate dehydrogenase
GFP: Green fluorescent protein
HA tag: Human influenza hemagglutinin tag
HBS: HEPES buffered saline
HEPES: 4-(2-Hydroxyethyl)-1-piperazineethanesulfonic acid
HRP: Horseradish peroxidase
IC: Immunocytochemistry
ID: Intracellular domain
Ig: Immunoglobulin-like
LAMP1: Lysosomal-associated membrane protein 1
min: Minute
NCAM2: Neural cell adhesion molecule 2
NCAM2ΔED: NCAM2 fragment comprising transmembrane and intracellular domains of NCAM2
NCAM2-VN: VN-tagged NCAM2
NCAM2ΔED-VN: VN-tagged NCAM2ΔED
PBS: Phosphate-buffered saline
PL: Proximity ligation
PMSF: Phenylmethylsulfonyl fluoride
PVDF: Polyvinylidene difluoride
siRNAs: Small interfering RNAs
SNPs: Single nucleotide polymorphisms
TBS: Tris-buffered saline
VC: C-terminal amino acid residues 155–238 of the fluorescent protein Venus
VN: N-terminal 155 amino acid residues of the fluorescent protein Venus
WB: Western blot
