# Supplementary material for: The BACE1-generated C-terminal fragment of the neural cell adhesion molecule 2 (NCAM2) promotes BACE1 targeting to Rab11-positive endosomes
Source: Cell Mol Life Sci. 2022 Oct 17;79(11):555. doi: 10.1007/s00018-022-04575-w (PMC9576659; doi:10.1007/s00018-022-04575-w)
Supplement: Supplementary file 1 — Supplementary file1 (PDF 5025 KB) [file 18_2022_4575_MOESM1_ESM.pdf]

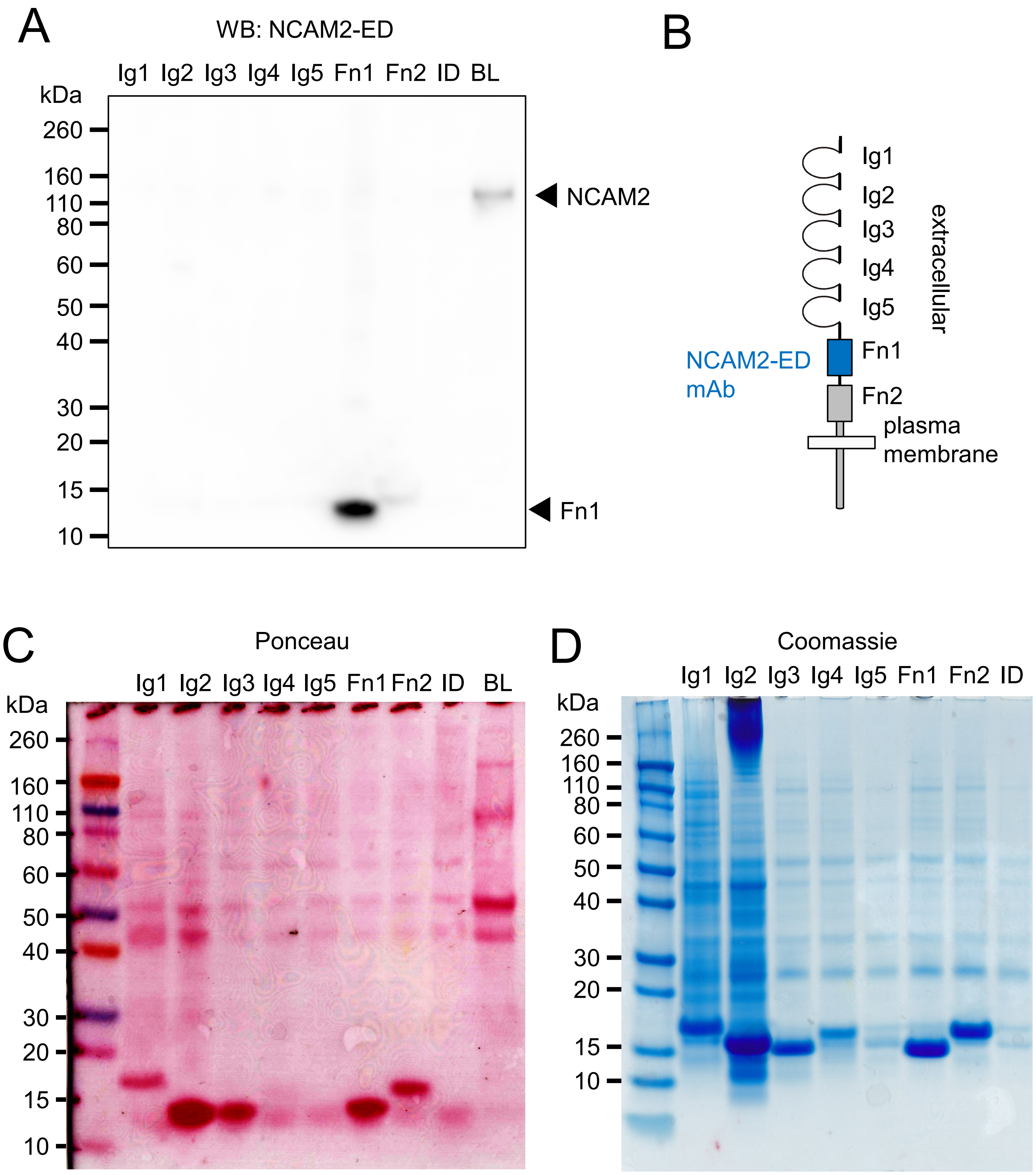

Supplementary Figure S1

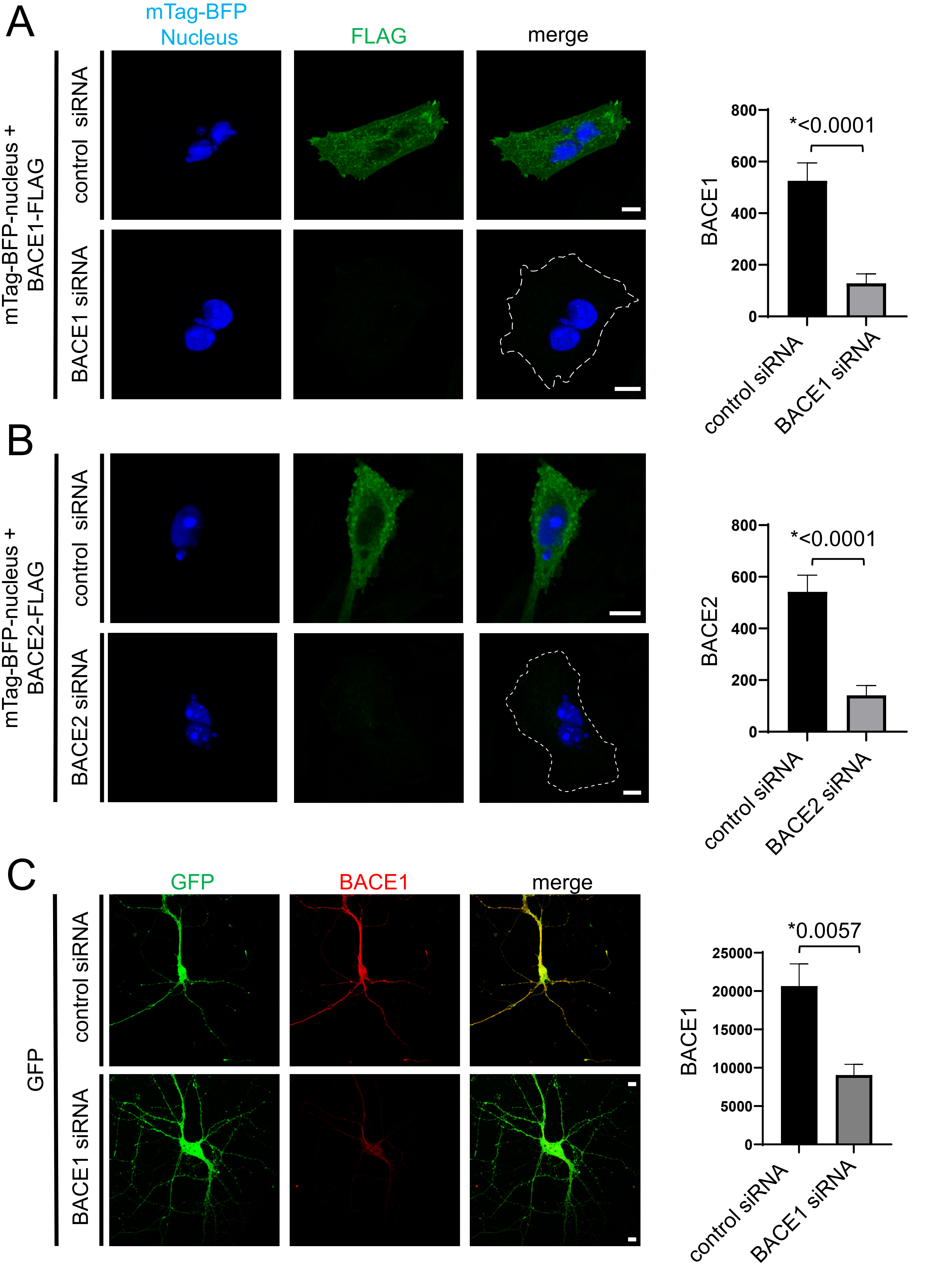

Supplementary Figure S2

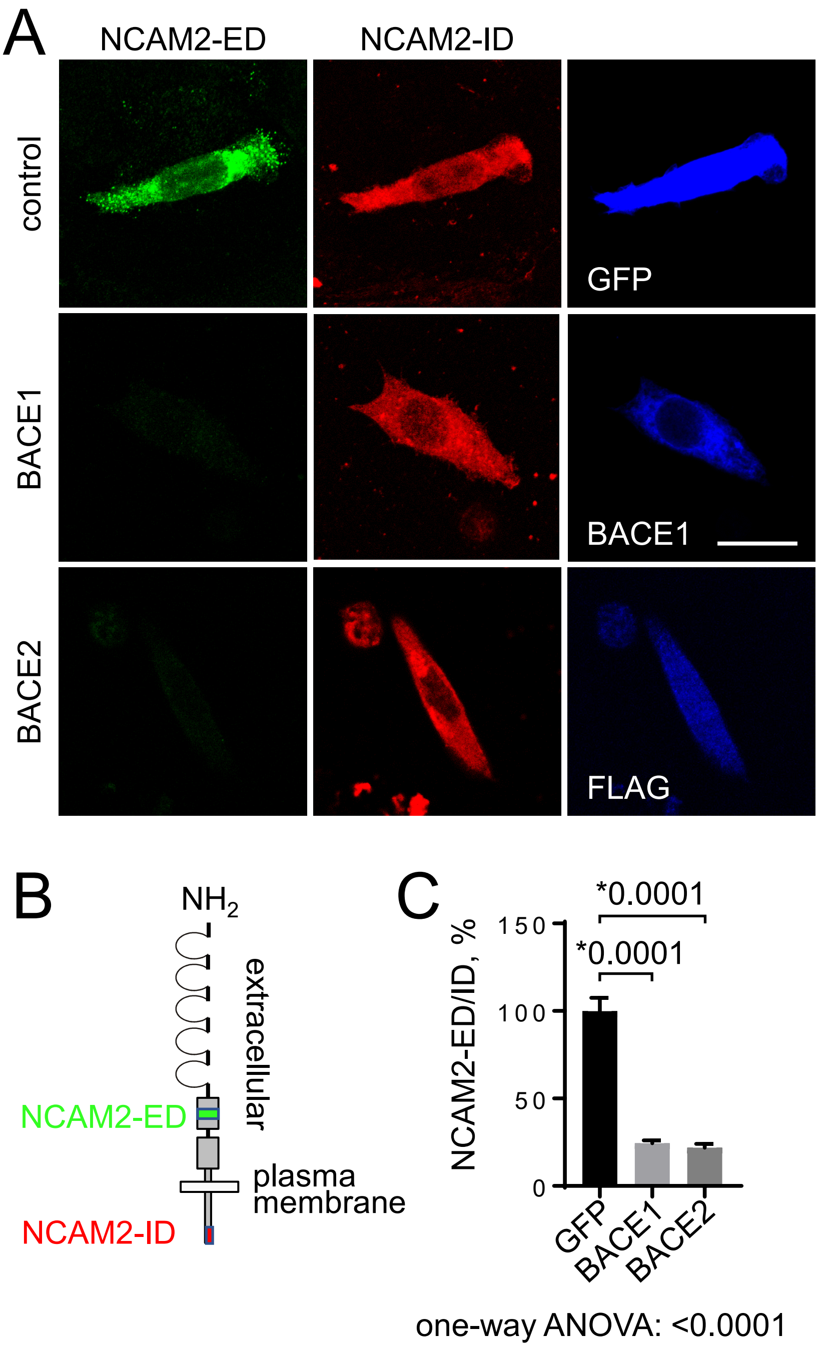

Supplementary Figure S3

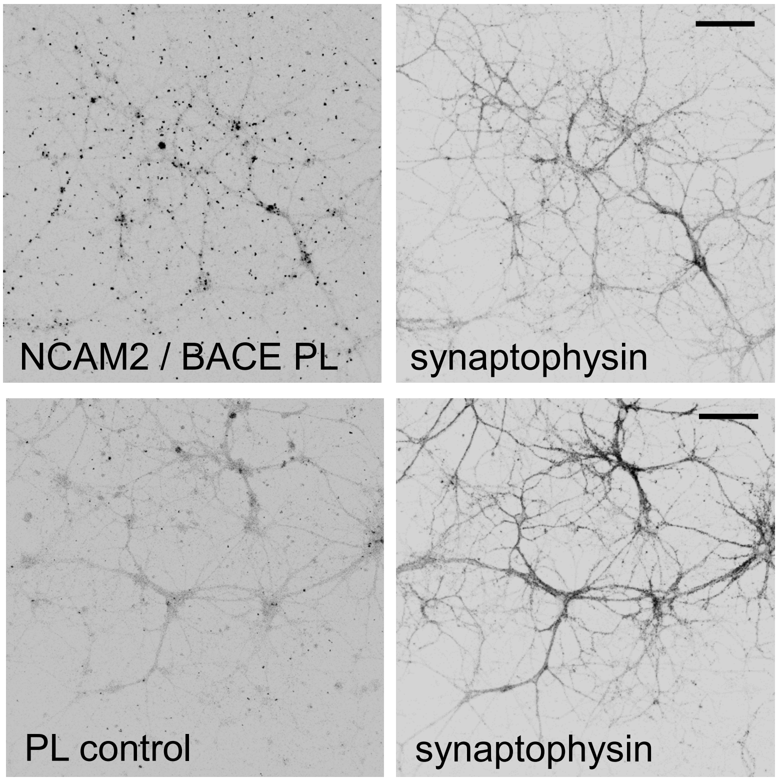

Supplementary Figure S4

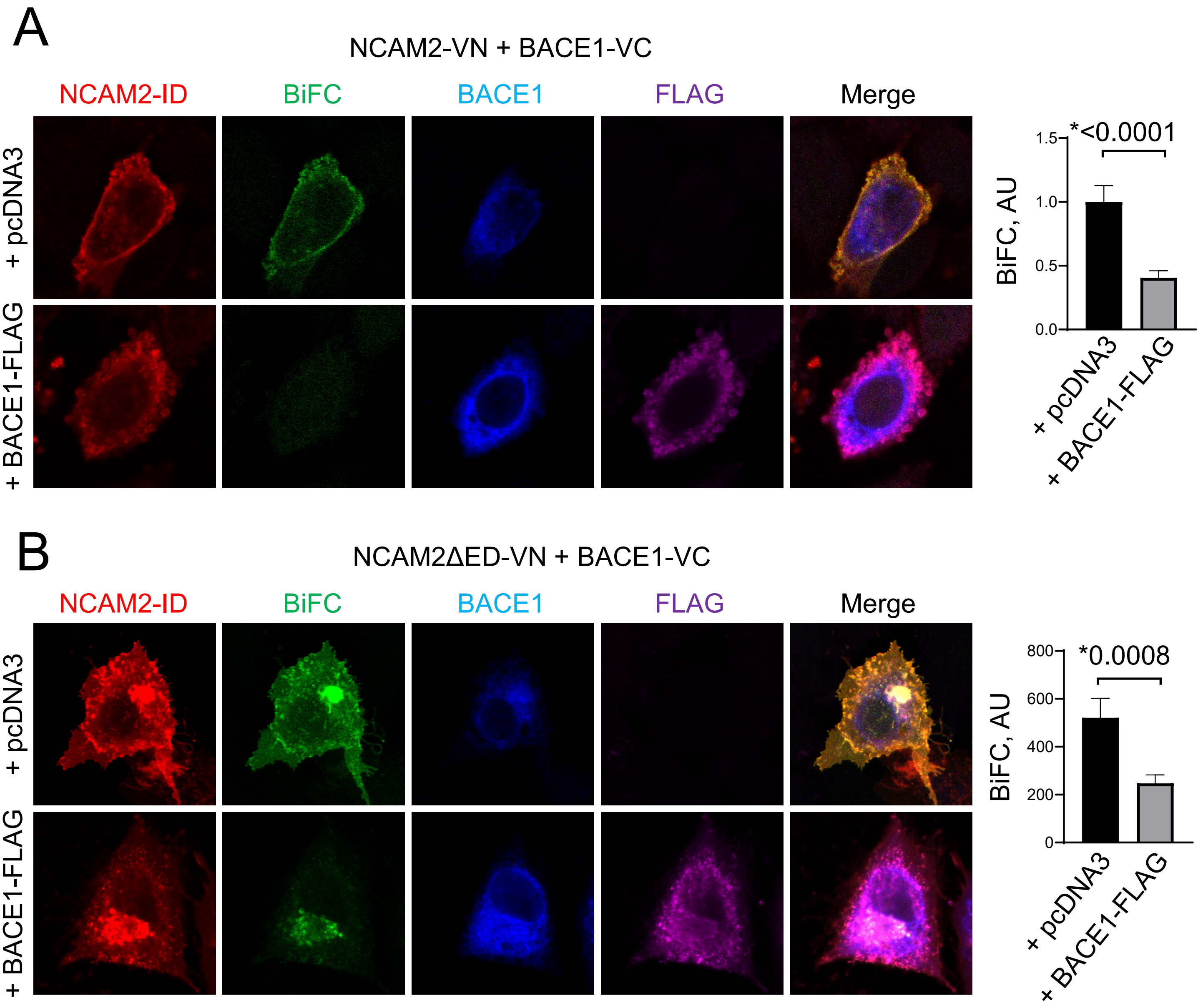

Supplementary Figure S5

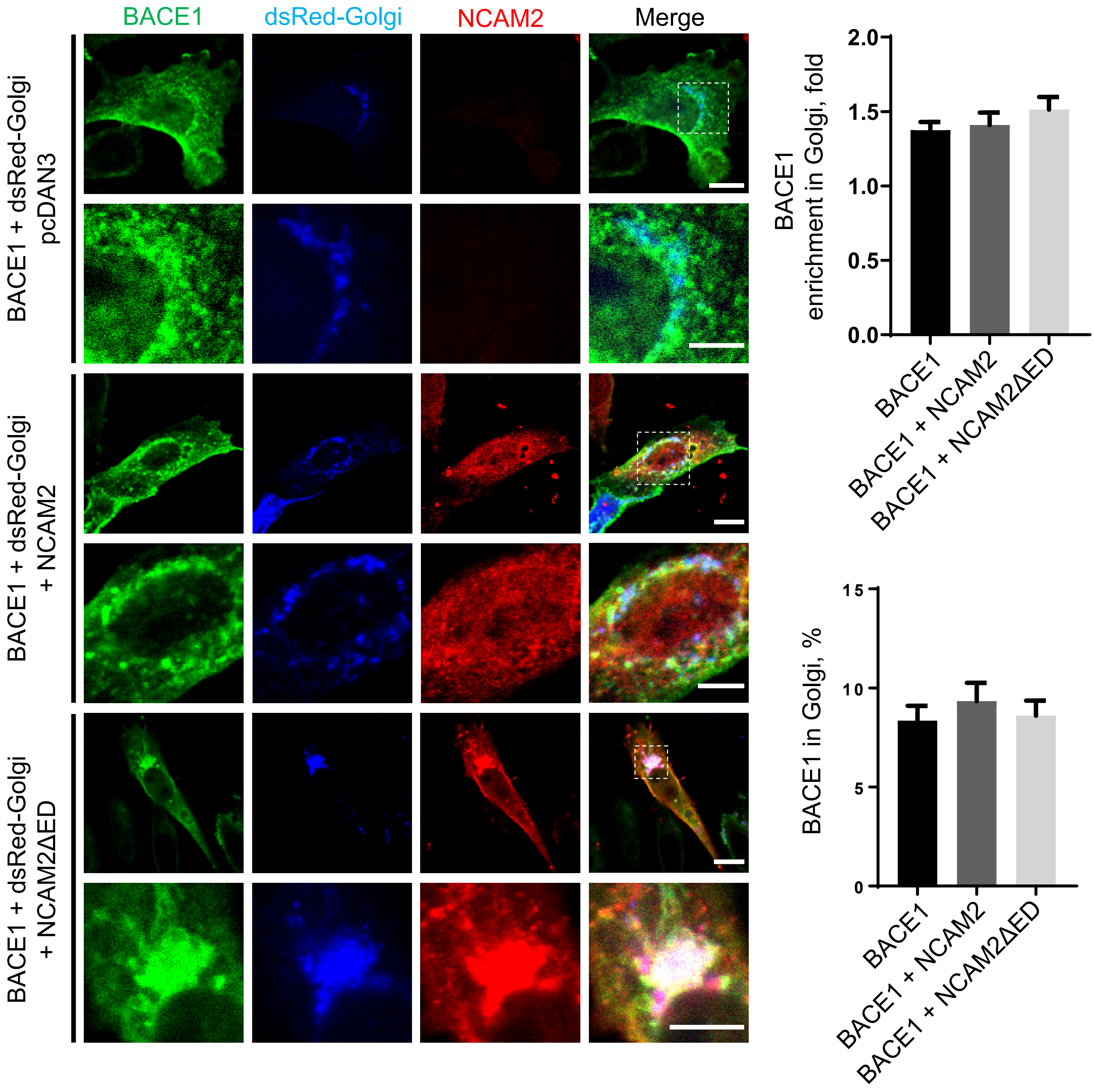

Supplementary Figure S6

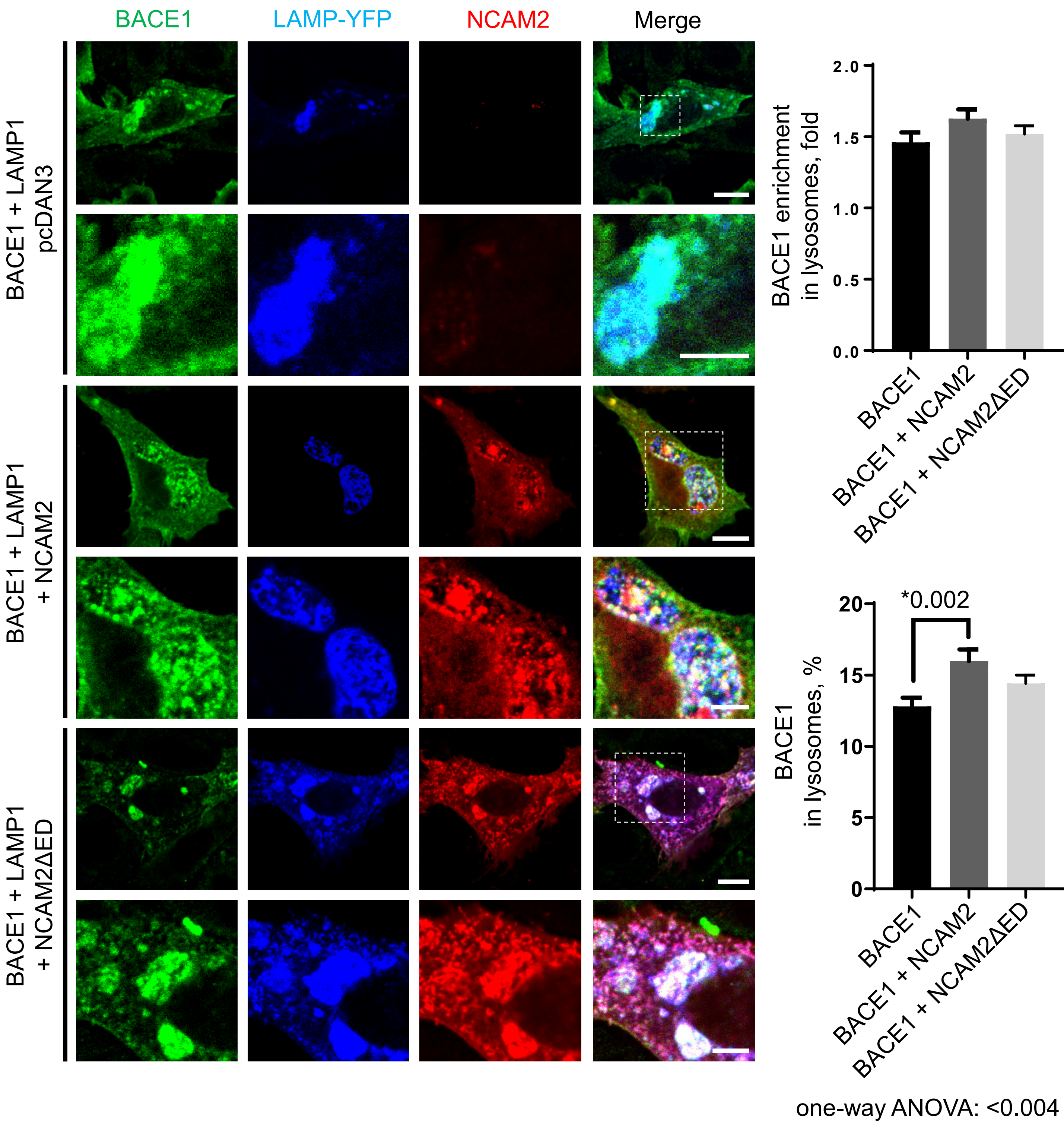

Supplementary Figure S7

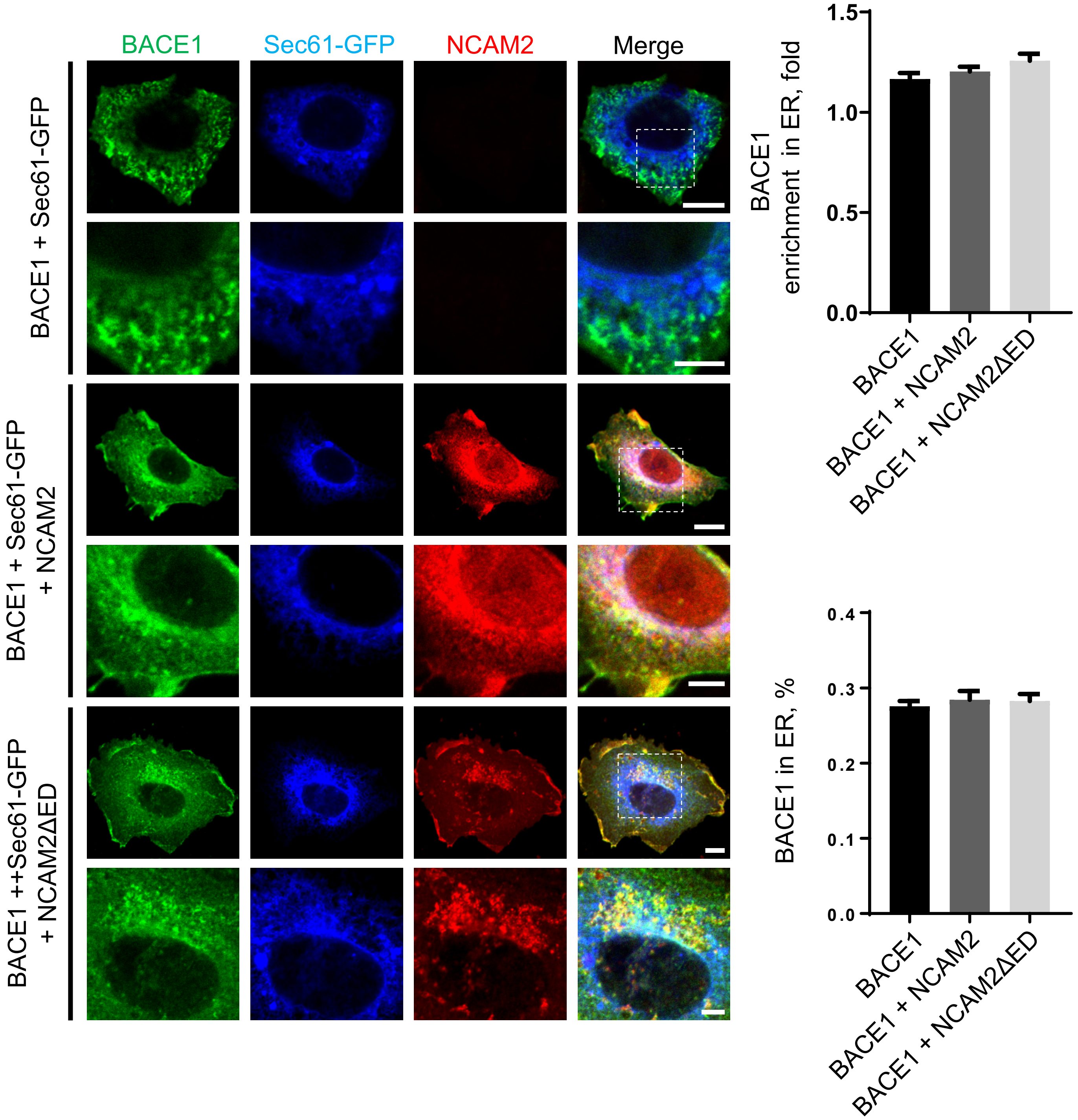

Supplementary Figure S8
